# Reduced Interhemispheric Coherence in Cerebellar Kainic Acid-Induced Lateralized Dystonia

**DOI:** 10.3389/fneur.2020.580540

**Published:** 2020-11-23

**Authors:** Elena Laura Georgescu Margarint, Ioana Antoaneta Georgescu, Carmen Denise Mihaela Zahiu, Stefan-Alexandru Tirlea, Alexandru Rǎzvan Şteopoaie, Leon Zǎgrean, Daniela Popa, Ana-Maria Zǎgrean

**Affiliations:** ^1^Division of Physiology and Neuroscience, Carol Davila University of Medicine and Pharmacy, Bucharest, Romania; ^2^Institut de biologie de l'Ecole normale supérieure (IBENS), Ecole normale supérieure, CNRS, INSERM, PSL Research University, Paris, France

**Keywords:** cerebellum, asymmetries, intra-cortical oscillations, dystonia, kainic acid, mice, movement disorder

## Abstract

The execution of voluntary muscular activity is controlled by the primary motor cortex, together with the cerebellum and basal ganglia. The synchronization of neural activity in the intracortical network is crucial for the regulation of movements. In certain motor diseases, such as dystonia, this synchrony can be altered in any node of the cerebello-cortical network. Questions remain about how the cerebellum influences the motor cortex and interhemispheric communication. This research aims to study the interhemispheric cortical communication between the motor cortices during dystonia, a neurological movement syndrome consisting of sustained or repetitive involuntary muscle contractions. We pharmacologically induced lateralized dystonia to adult male albino mice by administering low doses of kainic acid on the left cerebellar hemisphere. Using electrocorticography and electromyography, we investigated the power spectral densities, cortico-muscular, and interhemispheric coherence between the right and left motor cortices, before and during dystonia, for five consecutive days. Mice displayed lateralized abnormal motor signs, a reduced general locomotor activity, and a high score of dystonia. The results showed a progressive interhemispheric coherence decrease in low-frequency bands (delta, theta, beta) during the first 3 days. The cortico-muscular coherence of the affected side had a significant increase in gamma bands on days 3 and 4. In conclusion, lateralized cerebellar dysfunction during dystonia was associated with a loss of connectivity in the motor cortices, suggesting a possible cortical compensation to the initial disturbances induced by cerebellar left hemisphere kainate activation by blocking the propagation of abnormal oscillations to the healthy hemisphere. However, the cerebellum is part of several overly complex circuits, therefore other mechanisms can still be involved in this phenomenon.

## Introduction

The primary motor cortices play the most critical role in generating movement-related neural impulses and controlling the execution of the voluntary muscular activity, together with the cerebellum and basal ganglia (1). The synchronization of neural activity in the cerebello-cortical network is crucial for regulating complex movements (2, 3), even though some aspects of the cerebellar contribution to oscillatory brain activity remain still unclear. Brain connectivity can be assessed by estimating the extent to which two or more brain regions oscillations are similar (4). For this reason, one of the most often used methods to evaluate the brain's synchronous oscillations is coherence, a mathematical parameter.

The cerebral hemispheres inhibit one another through the corpus callosum (5–7). Transcranial magnetic stimulation (TMS) parameters such as the silent period (a pause in the electromyography during voluntary movement after TMS) can indicate the excitability of the corticospinal system (8). Silent period was found to be decreased in patients with focal hand dystonia and spasmodic dysphonia, indicating a diminished intracortical inhibition in dystonia (9). In patients with mirror dystonia (triggered by movements of the healthy arm), the interhemispheric inhibition was reduced (10). Also, reduced interhemispheric inhibition was found in patients with writer's cramp dystonia (11).

Dystonia is a movement disorder characterized by prolonged or intermittent muscle contractions that cause abnormal, often repetitive movements, postures, or both (12). Dystonic movements are often initiated or worsened by voluntary action and associated with overflow muscle activation (13). The most common form of primary dystonia is focal dystonia, with adult-onset involvement of a region of the body (e.g., neck in cervical dystonia, hand in writer's cramp). Writer's cramp is task-specific dystonia, occurring in patients having a long history of repetitive, stereotyped writing before the onset of dystonia (14). Dystonia is typically treated with botulinum toxin injections, which block the peripheral expression of the disease but not the anomalous motor commands generated in the central nervous system (15). There are clinical reports of pure cerebellar lesions producing dystonia in humans (16, 17), and dystonia has been associated with cerebellar atrophy (18). Therapeutic modulation of the cerebellum or its outflow pathway was occasionally found to be effective in the treatment of dystonia (19, 20). In animal models, dystonia can be triggered by kainic acid (a glutamate receptor agonist) injection into the cerebellum surface (21–23). Raike et al. showed that focal and generalized dystonia may have similar physiopathology and can be treated with similar strategies (24).

Here, we investigated the influence of the cerebellum on the interhemispheric motor cortices coherence in an animal model of focal cerebellar dystonia. We pharmacologically induced dystonia to adult male albino mice by administering low doses of kainic acid on the left cerebellar hemisphere. We combined motor behavior observations on *in vivo* recordings with concomitant electrocorticography (ECoG), electromyography (EMG) data, and computations of cortico-muscular and interhemispheric coherence before and during dystonia, for 5 consecutive days.

## Materials and Methods

This study was conducted in accordance with local and national policies for the good practice on animals used for scientific purposes, following the recommendations of the European Communities Council Directive 2010/63/EU, and with the approval of Ethical Committee for Animal Research of “Carol Davila” University of Medicine and Pharmacy, Bucharest, Romania (#PO-35-F-03).

### Animals

Experiments were carried out on 3 months old Swiss albino mice (n=13) weighing between 44 and 52 g. Mice were provided water and food *ad libitum* and housed on a 12 h light/dark cycle and room temperature.

### Electrode Insertion Surgery

**Figure 1:**
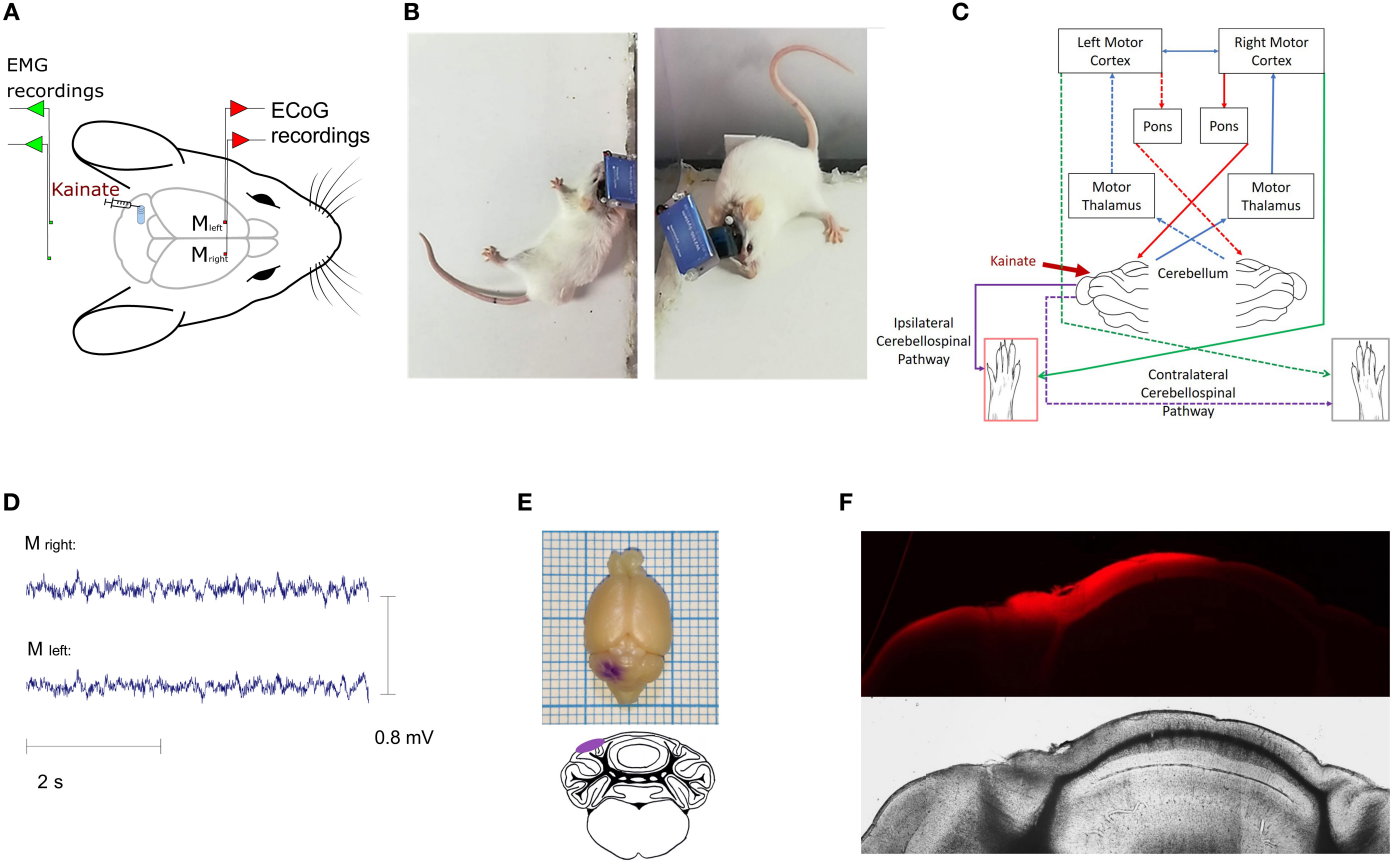
**(A)** Illustrative representation of the recording setting; placement of the ECoG electrodes for the (left and right) primary motor cortices and EMG electrodes for (left and right) nuchal region muscles; **(B)** Examples of dystonic attacks during a post-kainate recording session; **(C)** Schematic diagram of the cerebro-cerebellar loop with the indication of the kainate administration site. Dotted arrows illustrate the contralateral pathway; **(D)** Example of primary motor cortex ECoG recordings in the post-kainate state; **(E)** Gentian-violet staining through the cannula; **(F)** Fluorescence (Latex beads, λex ~538 nm; λem ~584 nm, Sigma–Aldrich) at the injection site indicating the kainate spread on the cerebellum cortex (Nikon epifluorescence microscope, 4× magnification).

Surgeries were performed under general isoflurane anesthesia (1.5–2% maintenance concentration and 4% induction concentration for 1–2 min) with adjustment following the level of response to the withdrawal pain reflex. Also, buprenorphine (50 μg/ kg) was subcutaneously injected for added pain control. Throughout the procedure, the mouse temperature was kept within physiological limits using a heating pad system (DC Temperature Controller, FHC). After a subcutaneous injection with local anesthetic (lidocaine, 1 ml, 2%), a median skin incision of the scalp and removal of subcutaneous tissue was performed. Craniotomies with a diameter of 1 mm were drilled at the level of the left and right motor primary cortices (2.2 mm anterior and 2.2 mm lateral to bregma) and also for positioning the ground and the reference electrode (2 mm posterior and 2 mm lateral to the right in relation to lambda). Nichrome ECoG and EMG electrodes (Kanthal, Palm Coast, FL, United States) were made from a flexible wire (0.15 mm) and placed on the dura mater's surface. Subcutaneously, two EMG electrodes built from the same wire were placed on the left and right muscles (one for each) of the nuchal region (Figure 1A). The electrodes were previously de-insulated at both ends (2 mm). For fixation, the electrodes were covered with dental cement (Pi-Ku-Plast HP 36, Bredent GmbH, Germany) and connected to the pins used for headstage attachment. An infusion cannula (C315GS-5 guide cannula, Plastics One, United States) was fixed on the dura mater surface 7 mm posterior and 2.4 mm left lateral to the bregma (over the left posterior lobe, Crus I) (Figures 1A–C). After implantation, the mice were left for 4 days to recover in the same room where they were to be recorded. Buprenorphine (50 μg/kg) was injected once a day for the next 3 days.

### Data Acquisition

For the ECoG and EMG recordings, we used a Multi-Channel Systems W2100 wireless interface board. An acquisition frequency of 1 kHz and a 4-channel W2100-HS4-opto Headstage (with a weight of 1.9 and 3.8 g for the battery) were used. The mice were allowed to move freely, and their behavior was video monitored by two cameras (Hero 3+ Silver, GoPro, United States), one placed at the level of the mouse and also a camera that recorded the movements of the mouse from above. On the first day of the experiment (baseline day), no kainic acid was administered. In the next five consecutive days, the recordings were made both before (10 min) and after (90 min divided into 3 blocks of 30 min) injecting kainic acid. Before applying kainic acid, the mice were briefly anesthetized with isoflurane (Rompharm, Romania). The recordings began 1 min after the mouse awoke. Also, we added a control group (*n* = 4) for which we replaced kainate solution with saline solution 0.9% using the same recording protocol.

### Video Recordings Monitored Motor Behavior of the Animals in an Open Field

On the first day of the experiment (baseline day), we recorded the mouse behavior together with electrophysiology signals *in vivo* without the administration of kainate. For the next five consecutive days, we applied 0.5 ± 0.1 μl of kainate (Sigma-Aldrich, 200 μg/ml, combined with 0.9% saline solution as a vehicle) (21, 23) directly on the surface of the left posterior cerebellar lobe, Crus I, through the chronically implanted cannula. The application was performed under brief anesthesia (2–3 min) with a C315I/20-49 internal cannula (Plastics One, United States) cut to fit the guide cannula with a 0.7 mm Projection. Dystonic attacks were identified and rated on-site and off-site using a previously published scale (23, 25), where 0 = normal motor behavior; 1 = abnormal motor behavior but no dystonic positions; 2 = minor motor impairment, dystonic-like positions when disturbed; 3 = moderate deficiency, many spontaneous dystonic positions; and 4 = severe impairment, prolonged dystonic postures. As the dystonic movements and postures were observed especially on the left part of the body, we assessed a score for hemidystonia (24). For each body part: head and neck, trunk, and limbs was assigned 1 if there were signs of dystonia, and 0 if there were not. A score was given for each 10-min epoch. We also analyzed the total time the mouse is exploring the arena as a percentage of the total time of recording [active wake % (AW%)] for kainate and control groups.

### Histology

To confirm the position of the cannula and to examine the spread of the kainate solution within the cerebellum, we histologically examined the brains. In order to mimic the administration of the same amount of kainate solution, we applied through the cannula a volume of 0.5 ± 0.1 μl fluorescent tracer (Latex beads, sulfate-modified polystyrene, fluorescent red, solids 2.5% found in aqueous solution, mean particle size 0.1 μm, fluorescence λex ~538 nm; λem ~584 nm, Sigma-Aldrich). Also, in order to identify the macroscopical spread of the substance on the cortical surface of the cerebellum, 0.5 ± 0.1 μl gentian violet (hexamethyl pararosaniline chloride) was injected through the cannula. The mice were then sacrificed and, after the formaldehyde perfusion, whole brains were removed from the skull and kept for another 24 h in formaldehyde. Brains were included in agarose (2%), then sliced into 90 μm coronal sections using a vibratome (Leica VT 1000S). Fluorescence microscopy images were taken using a Nikon epifluorescence microscope at 4x.

### Data Analysis

The artifacts were excluded from the analysis. Power spectral density values for left and right primary cortices and for left and right EMG were estimated with the Welch approach with 2 s windows overlapping 1 s from the 1 kHz signal with the MATLAB function *pwelch*. Coherence values were obtained using the MATLAB function *mscohere*, from power spectral density estimates of pairs of ECoG recordings Frequency bands were defined as follows: Delta: 0.5–3.5 Hz, Theta: 4–12,5 Hz; Beta: 13–30 Hz; Low-Gamma: 30,5–48 Hz; High-Gamma: 52–100 Hz and analyzed before and after kainate left cerebellar hemisphere administration. Interhemispheric coherence (between left and right primary motor cortices), coherence between right primary motor cortex (affected side) ECoG and left EMG, coherence between left primary motor cortex ECoG (unaffected side) and right EMG were calculated for all days of recordings. The real and imaginary coherences were determined for each band for the interhemispheric coherence to avoid contamination by volume conduction (26).

The power spectral density (0.5 to 250 Hz frequency range) of the EMG was separated into the following frequency bands: Delta: 0.5–3.5 Hz, Theta: 4–12,5 Hz; Beta: 13–30 Hz; Low-Gamma: 30,5–48 Hz; High-Gamma: 52–100 Hz; D5: 31.25–62.5 Hz; D4: 62.5–125 Hz; D3: 125–250 Hz (27). The electromyography analysis included the mean power frequency calculation for pre-kainate and post-kainate functional states. Mean frequency (MNF)

$$\begin{array}{l} MNF=\;\frac{\overset{M}{\underset{j=1}{\sum }}f_{j}P_{j}}{\overset{M}{\underset{j=1}{\sum }}P_{j}} \end{array}$$

is an average frequency calculated as the sum of product of the EMG power spectra and the frequency divided by the total sum of the power spectrum (28, 29). In the previous formula, *f*_*j*_ is the frequency value of EMG power spectrum at the frequency bin *j, P*_*j*_ is the EMG power spectrum at the frequency bin *j*, and *M* is the length of the frequency bin. This parameter provides information about how the power spectrum changes in time. Using MATLAB functions rms and abs, we calculated the root mean square (RMS) and average rectified value (μV).

After initial processing with MATLAB and Excel, the statistical analysis was completed with GraphPrism 6.0 using ANOVA test over time, Friedman test, Dunn's multiple comparisons test, +Tukey's multiple comparisons test, Mann Whitney test Wilcoxon matched-pairs signed-rank test and Kruskal–Wallis test after testing the normality distribution of the data, as appropriate. *P* < 0.05 were considered statistically significant.

## Results

### Mice Exhibited Dystonic Motor Behavior After Left Hemispheric Cerebellar Kainate Administration

Mice exhibited a dystonic-like behavior lateralized to the side of the injection (left) (Figures 1E,F) after the kainate application on the posterior cerebellar lobe, Crus I, especially in the left limbs (Figures 1A–C).

**Figure 2:**
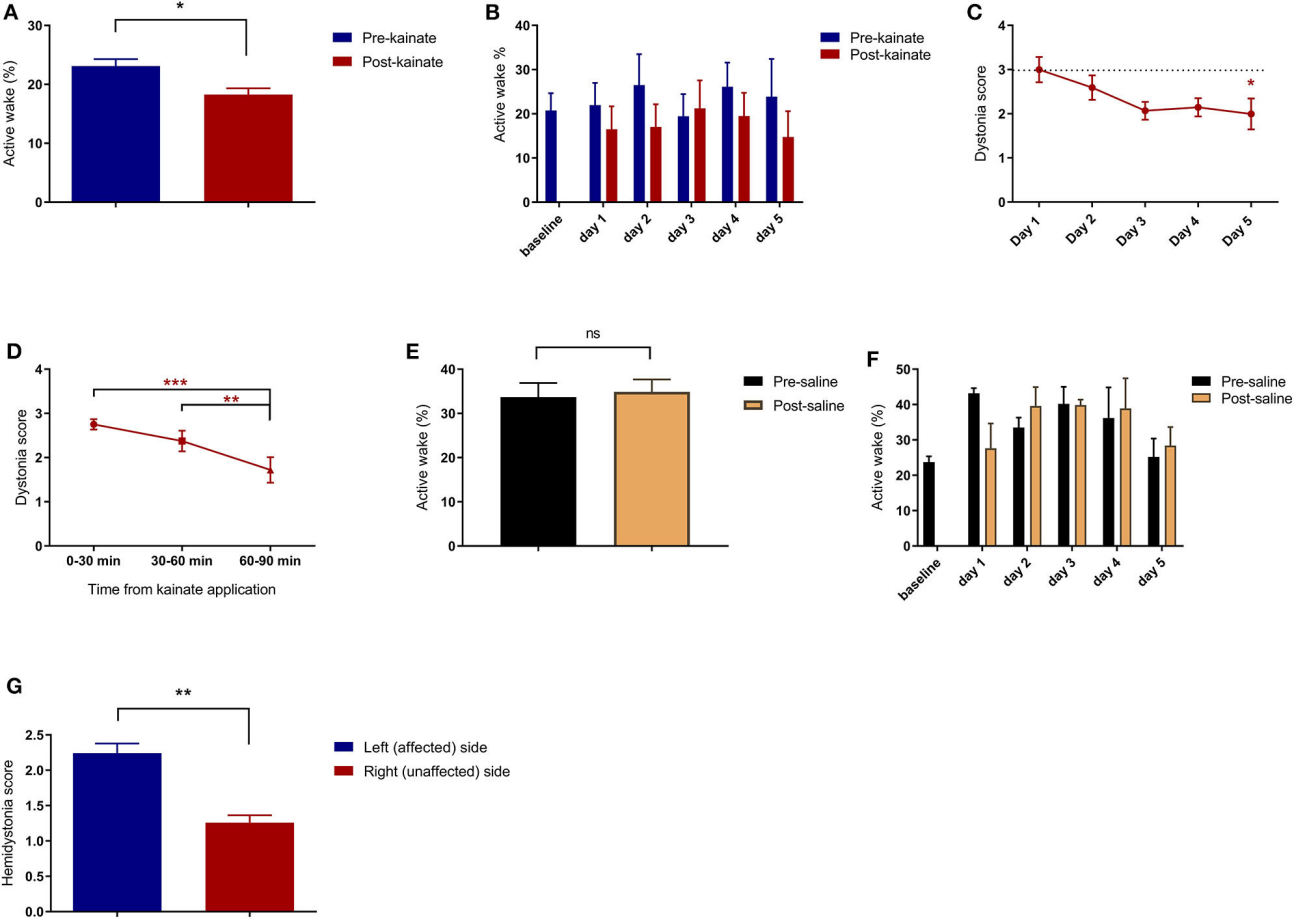
**(A)** Average of pre-kainate and post-kainate active wake percentages of all days of recordings ± SEM; **(B)** Average pre-kainate and post-kainate active wake percentages ± SEM from baseline day to day 5 of administration; **(C)** Average dystonia score per day of administration ± SEM, every day against baseline day; **(D)** Average dystonia score per 30-minute interval from kainate application ± SEM. Findings are expressed as average ± standard error of the mean (SEM) (*p < 0.05, **p < 0.01, and ***p < 0.001, Supplementary Table 1). **(E)** Active wake (%) in the control group; **(F)** Active wake (%) in the control group per day of dystonia; **(G)** Hemidystonia score for affected and non-affected sides.

The ECoG recordings for the left and right motor primary motor cortices indicated no epileptiform waves during the dystonic attacks (Figure 1D). The dystonic behavior started after a few minutes from the kainate application and consisted of lateralized prolonged contraction of the limbs, neck and trunk, tail, or extension of the limbs on the left side followed by periods of reduction of the symptoms (Supplementary Video 1 with simultaneous EMG recordings in Supplementary Figures 2, 3, Supplementary Video 2 shows pre-kainate behavior). We also observed a generalized reduction of movements and time of exploring the open field. When assessing the average active wake percentages (AW%) of all pre-kainate vs. post-kainate recordings, we observed a significant reduction in the total time spent walking after the kainate application (Figure 2A; Supplementary Table 1). Across days, we observed an upward trend of the AW% in the pre-kainate state when compared with the baseline day, but the differences were not significant (Figure 2B; Supplementary Table 1).

A dystonia score was allocated for every 10 min, and an average was calculated for every 30-min block from the kainate application. We observed a high average score in the first 30 min, followed by a significant decrease in the next 30 and 60 min (Figure 2D; Supplementary Table 1). Also, the average dystonia score per day decreased consistently from the first day of kainate administration until day 5 when the decrease was significant (Figure 2C; Supplementary Table 1). These results indicate that the severity of dystonia decreases with repeated application of kainate, suggesting a possible compensation mechanism or receptors becoming less sensitive. The hemidystonia score indicated a significantly increased score on the left (affected) side when compared to the right side (Figure 2G). No differences were observed between the pre- and post-saline recordings (control group, *n* = 4) (Figures 2E,F).

### Electromyography Shows Higher Muscular Activity After Left Hemispheric Cerebellar Kainate Administration

**Figure 3:**
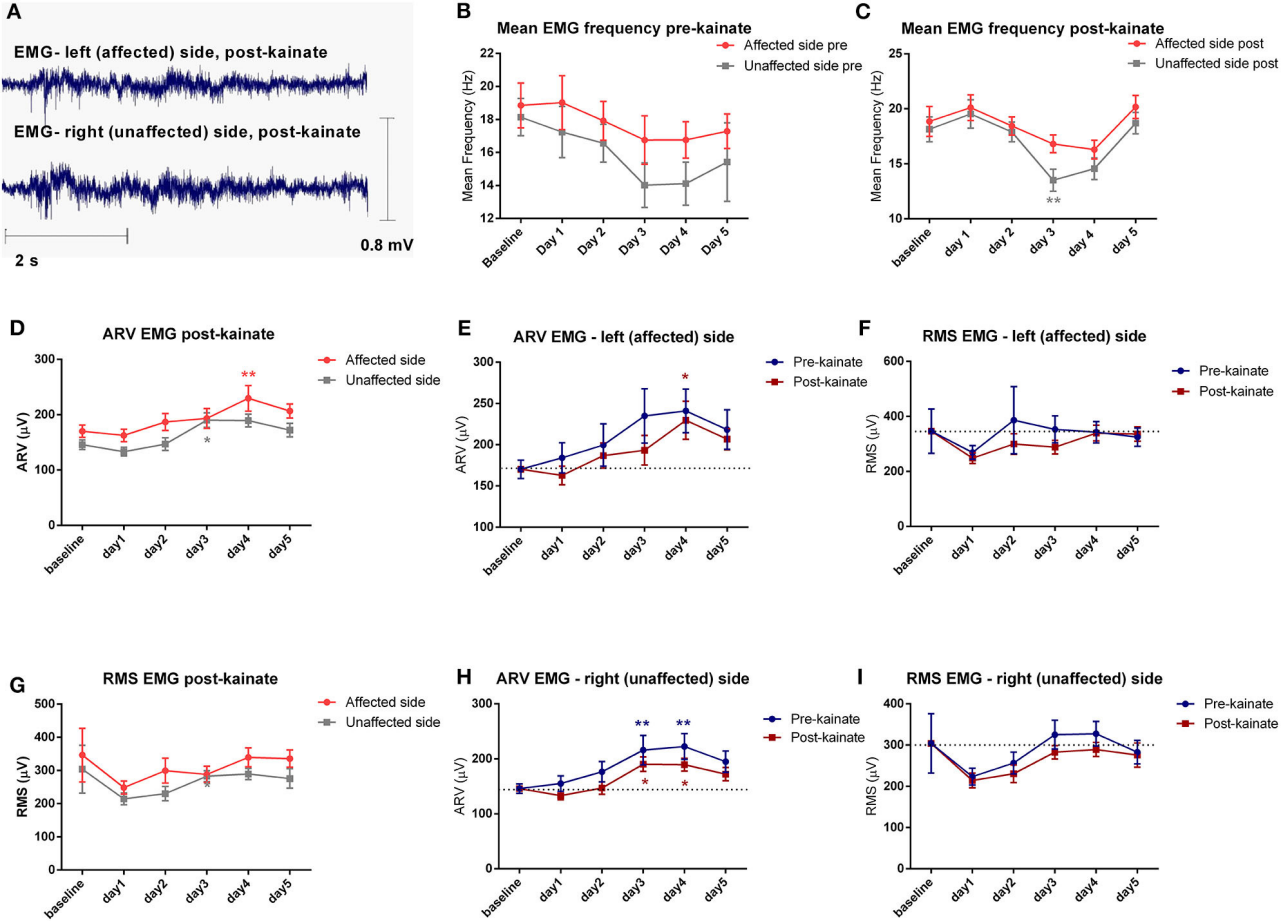
**(A)** Examples of raw EMG recordings during the dystonic attack on the left (affected) side and on the right (unaffected) side of the nuchal region; **(B)** Evolution by the day of kainate administration of the Mean Frequency (± SEM) of the EMG power spectra of the affected and unaffected side, recordings in the pre-kainate state; **(C)** Evolution by the day of kainate administration of the Mean Frequency of the EMG power spectra of the affected and unaffected side; **(D)** Affected side (left) vs. unaffected side (right) ARV EMG post-kainate evolution by the day of kainate administration; **(E)** Average rectified value (ARV) of the affected side during each day of experiment (μV); **(F)** Root mean square (RMS) of the affected side during each day of experiment (μV); **(G)** Affected side (left) vs. unaffected side (right) RMS EMG post-kainate evolution by the day of kainate administration; **(H)** Average rectified value (ARV) of the unaffected side during each day of experiment (μV); **(I)** Root mean square (RMS) of the unaffected side during each day of experiment (μV); Findings are expressed as average ± standard error of the mean (SEM), each day against baseline day (*p < 0.05 and **p < 0.01, Supplementary Table 2).

The analysis of the electromyographic recordings of the neck muscles indicated a tonic pattern during the dystonic outbreaks (Figure 3A). The mean frequency had a significant decrease for the unaffected side in day 3, followed by an increase to day 5 (Figure 3C). The values of the affected side followed a similar trend but remained higher in all days of kainate administration for the affected side. When we compared the mean frequency of the recordings in the pre-kainate state, the results followed a similar trend, but they were not significant (Figure 3B). Also, the mean frequency of the EMG power spectra was higher for the affected side, in all days.

To estimate the EMG amplitude, we calculated the average rectified value (ARV) (Figures 3D–H) and the root mean square (RMS) (Figures 3F–I). The average rectified value of EMG (ARV) had insignificant changes, with a tendency to increase to the maximum values in days 3 and 4, and then decrease slightly in day 5, on both EMG channels. The other amplitude estimator of EMG signal, the average root mean square (RMS), decreased significantly after kainate administration in the day 4 and day 5 for the left (affected) nuchal muscles, and in the day 2 for the right (unaffected) nuchal muscles.

### EMG Spectrum (Left Side), Motor Cortex Power Spectrum (Right Side), and Cortico-Muscular Coherence (Right-Left) of the Affected Side After Left Cerebellar Hemispheric Kainate Administration

**Figure 4:**
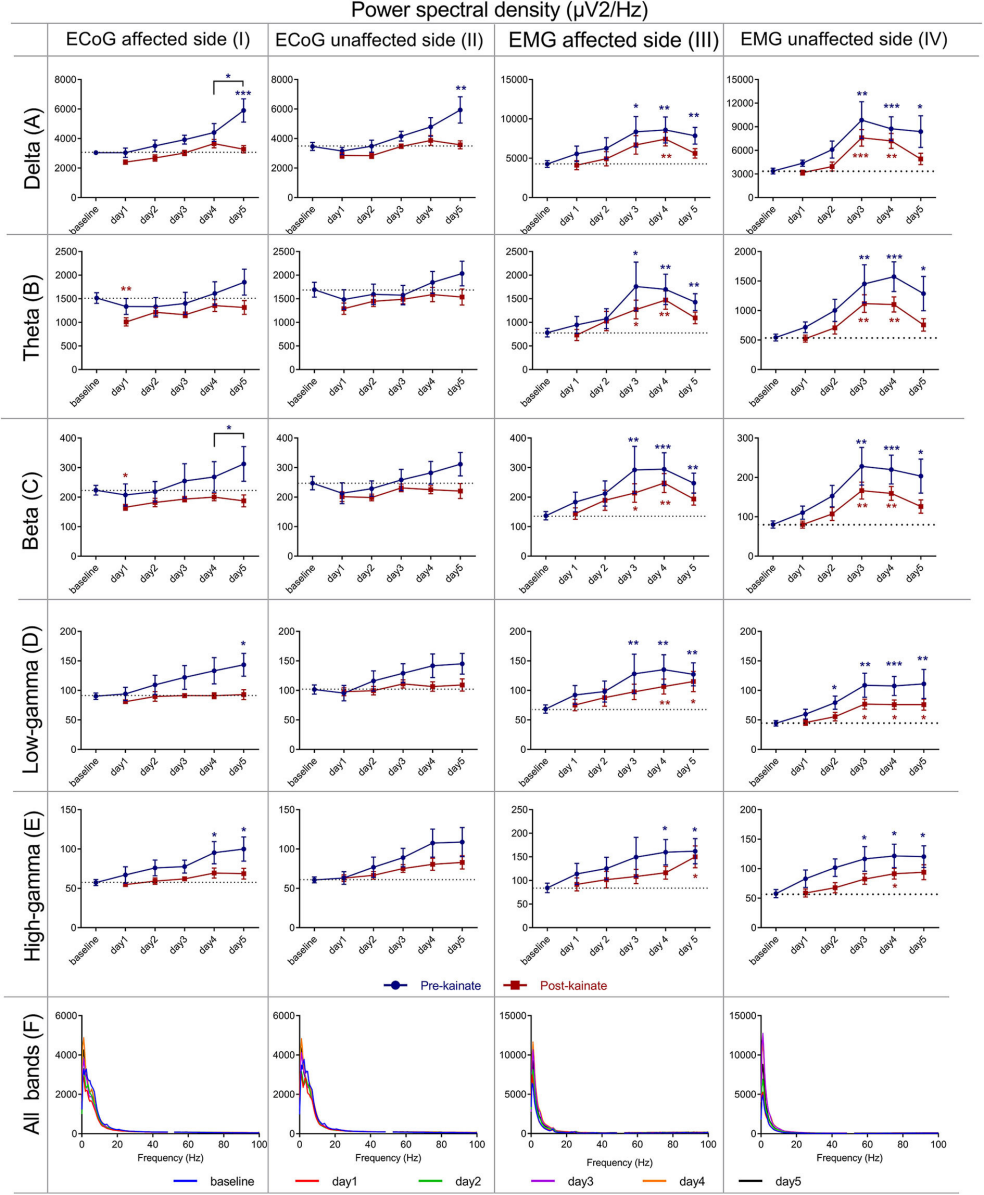
Power spectral density of the right (affected side) primary motor cortex ECoG across different frequency bands: **(IA)** Delta (0.5–3.5 Hz); **(IB)** Theta (4–12,5 Hz); **(IC)** Beta (13–30 Hz); **(ID)** Low-gamma (30.5–48 Hz); **(IE)** High-gamma (52–100 Hz) frequency bands across days of left cerebellar hemisphere kainate administration; **(IF)** Average ECoG Power Spectral Density of the right primary motor cortex across days of kainate administration. Power spectral density of the right (affected side) primary motor cortex ECoG across different frequency bands **(IIA)** Delta (0.5–3.5 Hz); **(IIB)** Theta (4–12,5 Hz); **(IIC)** Beta (13–30 Hz); **(IID)** Low-gamma (30.5–48 Hz); **(IIE)** High-gamma (52–100 Hz) frequency bands across days of left cerebellar hemisphere kainate administration; **(IIF)** Average ECoG Power Spectral Density of the right primary motor cortex across days of kainate administration. Power spectral density of the (left) affected side EMG across different frequency bands **(IIIA)** Delta (0.5–3.5 Hz); **(IIIB)** Theta (4–12,5 Hz); **(IIIC)** Beta (13–30 Hz); **(IIID)** Low-gamma (30.5–48 Hz); **(IIIE)** High-gamma (52–100 Hz) frequency bands across days of left cerebellar hemisphere kainate administration; **(IIIF)** Average left EMG Power Spectral Density evolution across days of kainate administration. Power spectral density of the (right) unaffected side EMG across different frequency bands; **(IVA)** Delta (0.5–3.5 Hz); **(IVB)** Theta (4–12,5 Hz); **(IVC)** Beta (13–30 Hz); **(IVD)** Low-gamma (30.5–48 Hz); **(IVE)** High-gamma (52–100 Hz) frequency bands across days of left cerebellar hemisphere kainate administration; **(IVF)** Average left EMG Power Spectral Density evolution across days of the experiment. Spectra are expressed as average ± the standard error of the mean (SEM), each day against baseline day (*p < 0.05, **p < 0.01, and ***p < 0.001, Supplementary Tables 3, 4, 6, 7).

We found significant increases from day 3 of the power spectral density in all frequency bands of the EMG spectrum except for the bands D3, D4, and D5, both before and after kainate administration (Figures 4IIIA–F, Supplementary Figures 1A–E). In the low-frequency bands (delta, theta) and the beta band (Figures 4IIIA–C), there was a maximum increase of power spectral density in days 3 and 4, followed by a decrease in day 5. There was a continuous increase in gamma bands from day 1 to day 5 compared to baseline day (Figures 4IIID,E). Kainate administration reduced the power spectral density in all frequency bands when comparing the pre-kainate EMG recordings (Figures 4IIIA–E), with the post-kainate recordings in the left nuchal muscles.

The data assessed from the right motor cortex revealed a progressive increase compared with baseline in all ECoG bands, for the pre-kainate recordings (Figures 4IA–F). This incremental trend peaked on day 5 when the changes were significant in the delta and low gamma and day 4 and 5 for high gamma. When comparing values on day 4 to those recorded on day 5, we found a statistically significant increase for delta and beta bands. The post-kainate values significantly decreased in day 1 in comparison with the baseline in theta and beta bands. An incremental trend was also observed, and a reached a maximum was reached on day 4 for all bands, except for low gamma, whose highest values were recorded on day 5.

**Figure 5:**
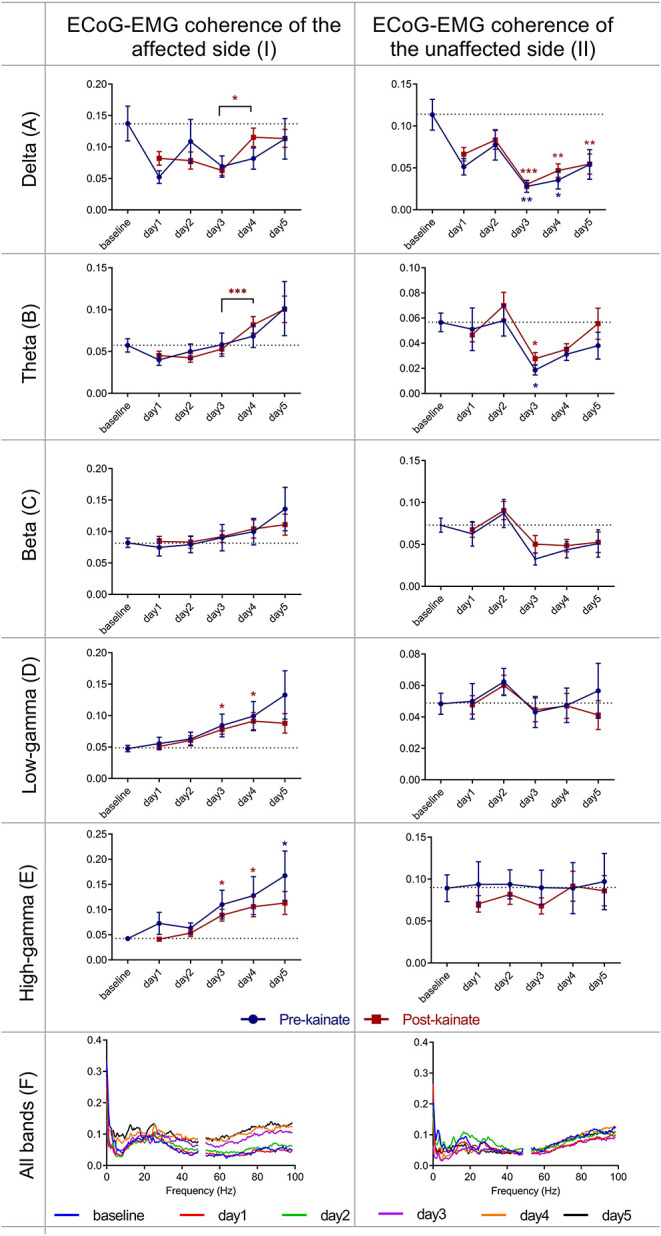
Coherence between right primary motor cortex ECoG and left EMG (affected side) and between left primary motor cortex ECoG and right EMG (unaffected side), across different frequency bands: **(IA)** Delta (0.5–3.5 Hz); **(IB)** Theta (4–12,5 Hz); **(IC)** Beta (13–30 Hz); **(ID)** Low-gamma (30.5–48 Hz); **(IE)** High-gamma (52–100 Hz) frequency bands across days of left cerebellar hemisphere kainate administration; **(IF)** Average coherence between right primary motor cortex ECoG and left EMG across days of the experiment. Coherence between left primary motor cortex (unaffected side) ECoG and right EMG across different frequency bands **(IIA)** Delta (0.5–3.5 Hz); **(IIB)** Theta (4–12,5 Hz); **(IIC)** Beta (13–30 Hz); **(IID)** Low-gamma (30.5–48 Hz); **(IIE)** High-gamma (52–100 Hz) frequency bands across days of left cerebellar hemisphere kainate administration; **(IIF)** Average ECoG–EMG coherence between the left primary motor cortex and right EMG across days of the experiment. Results are expressed as average ± the standard error of the mean (SEM), each day against baseline day (*p < 0.05, **p < 0.01, and ***p < 0.001, Supplementary Tables 5, 8).

Since the left part of the body was involved in dystonic postures ipsilateral to the left cerebellar cortex where the injection site of kainate was made and then the cerebellum sends projections to the contralateral (right) motor cortex, we investigated the coherence between the right motor cortex ECoG and the left part of the neck muscles EMG (Figures 5IA–F). The affected side ECoG–EMG coherence in the delta band tended to decrease starting the first day of the experiment. In theta band, the coherence decreased in the first 2 days of the experiment compared to baseline day and increased in day 4 and day 5 compared to baseline day, both before and after kainate administration. In the beta band, we found an increase of coherence in the last 3days of the experiment after kainate administration, both before and after kainate. These changes in delta, theta, and beta were not significant. In gamma bands (low and high), cortico-muscular coherence increased gradually from day 1 until day 5 compared to baseline day; the change is significant in day 3 and day 4 after kainate injection. Similarly, the recordings of the cortico-muscular coherence before kainate administration in gamma bands increased continuously until day 5 when the change was significant for the high gamma band, compared to baseline values.

### EMG Spectrum (Right Side), Motor Cortex Power Spectrum (Left Side) and Cortico-Muscular Coherence (Left-Right) of the Unaffected Side After Left Cerebellar Hemispheric Kainate Administration

In the power spectrum density of the right (unaffected) side EMG (Figures 4IVA–F, Supplementary Figures 1B–F), we found significant increases starting from day 2 (low and high gamma) or day 3 (delta, theta, beta) of kainate administration in all frequency bands, except for the bands D3, D4, D5, both before, and after kainate administration. In the delta, theta, and the beta band, there was a maximum increase of power spectral density in the days 3 and 4, followed by a decrease in day 5 for both pre- and post-kainate recordings. There was a continuous increase in gamma bands from day 1 to day 3 with a plateau until day 5 for the pre- and post-kainate data. All days were analyzed in comparison to the baseline day. Similar to left side EMG data, kainate administration reduced the power spectral density in all frequency bands when comparing the pre-kainate EMG recordings.

For the left motor cortex (Figures 4IIA–F), the analysis of assessed data showed power spectral density increase in all ECoG motor cortex bands for both pre- and post-kainate recordings, but significant only for the delta band, in the pre-kainate state during day 5. Changes were visible from the first day of the kainate application. All values peaked on day 5 in the pre-kainate recordings, except for low-frequency bands recorded in the post-kainate state with their maximum values appear in day 4 for delta and theta and day 3 for the beta. Also, in all the bands, the post- kainate powers were lower than in the pre-kainate.

ECoG–EMG coherence of the unaffected side (Figures 5IIA–F) showed significant changes only in delta and theta bands, with minimum changes in the other bands. Delta band power decreased significantly in day 3, 4 (for both pre- and post-kainate), and 5 of kainate administration (post-kainate). The coherence values remained <0.15 in all frequency bands.

### Interhemispheric Primary Motor Cortices Coherence

**Figure 6:**
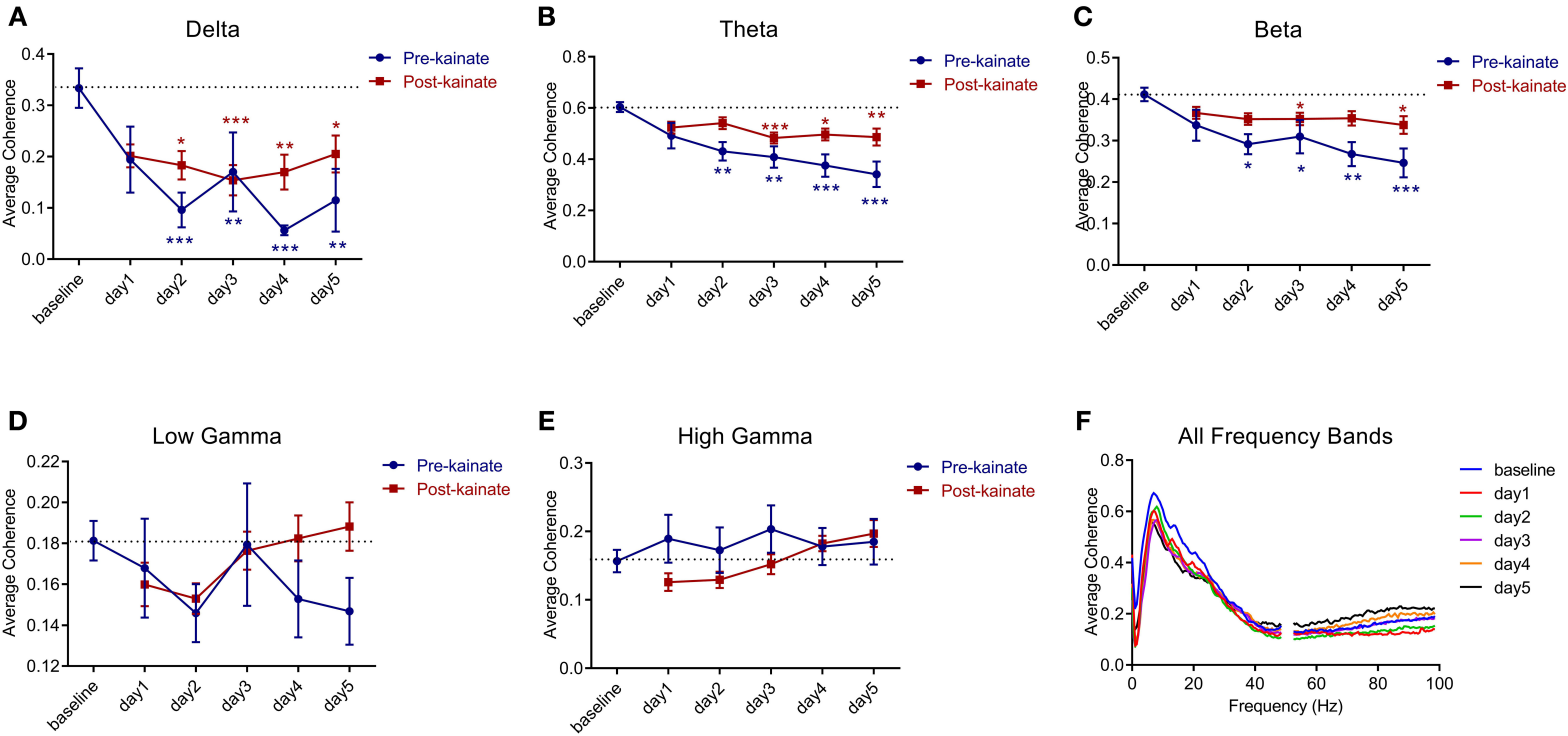
Coherence between left and right primary motor cortices' ECoGs across different frequency bands: **(A)** Delta (0.5–3.5 Hz); **(B)** Theta (4–12.5 Hz); **(C)** Beta (13–30 Hz); **(D)** Low-gamma (30.5–48 Hz); **(E)** High-gamma (52–100 Hz) frequency bands across days of left cerebellar hemisphere kainate administration; **(F)** Average interhemispheric coherence across days of kainate administration. Results are expressed as average ± the standard error of the mean (SEM), each day against baseline day (*p < 0.05, **p < 0.01, and ***p < 0.001, Supplementary Table 9).

**Figure 7:**
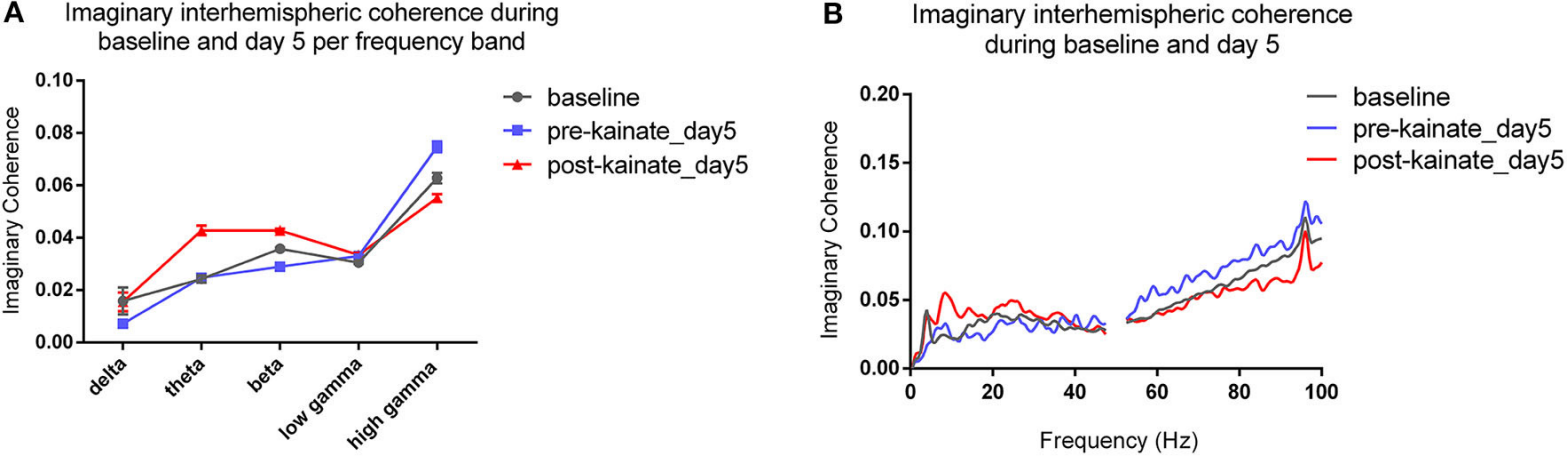
**(A)** Imaginary interhemispheric coherence during baseline and day 5 (pre- and post-kainate) per frequency band. Results are expressed as average ± the standard error of the mean (SEM), each day against baseline day; **(B)** Imaginary interhemispheric coherences across 0.5–100 Hz frequencies (average).

The interhemispheric coherence (Figures 6A–F) between left and right primary motor cortices shows a significant reduction in the delta, theta, and beta bands. The pre-kainate interhemispheric coherence shows significant reduction throughout the experiment in the delta, theta, and gamma bands starting with day 2 of kainate administration. The post-kainate data shows a decremental trend that is statistically significant from day 2 to day 5 in the delta, day 3 to day 5 in theta, and day 3 and 5 in the beta band. To avoid contamination by volume conduction, we calculated the imaginary part of coherence (26, 30). We discovered an apparent imaginary coherence coupling in low frequencies and in gamma that excludes volume conduction (Figures 7A,B). An increased imaginary part of coherence post-kainate was found in delta, theta, beta, results that were consistent with the real part of the coherence.

## Discussion

This research aims to examine the changes in the interhemispheric cortical communication between the primary motor cortices during normal and dystonic state after left hemispheric cerebellar activation with kainate. Similarly to previous studies, we showed that a limited region of cerebellar dysfunction could induce isolated abnormal movements such as limb prolonged contraction that resemble focal dystonia (24). The kainate microinjections into the left cerebellar cortex induced, especially ipsilateral dystonic contractions, whereas vermis microinjections induced generalized dystonia (21). After repeated kainic acid application on the left cerebellar hemisphere, we found a significant reduction of the interhemispheric coherence in low-frequency bands (delta, theta, beta). This change might indicate apparent cortical compensation for the initial disturbances. The lateralized dystonia lasted for at least 90 min after the injection, and the dystonia severity could be objectified in the form of a reduced general locomotor activity and a high score of dystonia. The motor condition of the mice was affected the most during the first 30 min after the injection and improved with time in the same day and also until day 5 as a possible adaptation or receptor desensitization. Forelimb and hindlimb were shown to be mapped in Crus I in monkeys (31), and also lobular structure studies of macaque, marmoset, rat, and mouse indicated that human Crus I and II are correspondent to Crus I in rodents (32). However, the cerebellar cortex displays a pattern of patches of functional units distributed mosaically, with multiple representations of the same body regions (33), which can explain the off-target effects observed in our experiments. Thus, our results might have been influenced not only by the spreading of the kainic acid at the injection site but also by the specific somatotopy of the cerebellar cortex.

Besides, we found a progressive increase in the power bands of the affected motor cortex spectra in both pre-and post-kainate conditions. EMG of the affected side had an increased power in all bands, with pre-kainate power having higher values than post-kainate ones. Even though the implant of the electrodes at the level of cerebral cortex or other experimental procedures might have impacted the neuronal activity in the pre-kainate states, the changes in coherence and power spectrum observed in these recordings were significant only after 2 or 3 days of kainate administration, suggesting a plastic reorganization or a form of compensation for the existing aberrant cerebellar activity.

The cortico-muscular coherence had a significant increase in gamma bands in days 3 and 4. In the contralateral side, EMG also has an increase in all bands, but power spectra did not change significantly. The cortico-muscular coherence suffered a decrease only in delta and theta bands (from day 3). In conclusion, cerebellar dysfunction during dystonia causes a loss of connectivity in the motor cortices, which may block propagation of abnormal oscillations.

### Asymmetries in Connection With Cerebellum Disturbances

While a large amount of data indicates a cerebellar involvement in dystonia, very few studies have investigated its involvement in brain dynamics. Using Positron Emission Tomography (PET), Rascol et al. (34) show that during the execution of a finger to thumb opposition motor task, these PD patients that are off medication exhibit a significant overactivation in the ipsilateral, but not in the contralateral cerebellar hemisphere. This finding suggests that off medication PD patients may try to compensate for their basal ganglia-cortical loop dysfunction using other motor pathways involving cerebellar relays. Moreover, Hanakawa et al. (35) used single-photon emission computed tomography to show that during gait on a treadmill, PD patients reveal inter-hemispheric asymmetries of cerebral blood flow. In particular, the hypoactivity only in the left cerebellar hemisphere, in contrast to the hyperactivity in the bilateral vermis, could be associated with the loss of lateral gravity shift in parkinsonian gait.

### Asymmetries in Focal Dystonia

Recent studies showed functional and structural changes in the cerebellum of patients with adult-onset primary focal dystonia, especially in the inferior olive, cerebellar nuclei, and cerebellar cortex (36, 37). Researchers revealed that people affected by cervical and hand dystonia had impaired reactions in a classical eyeblink conditioning paradigm. This reflex is linked to the olivo-cerebellar pathway and does not require cerebral and basal ganglia involvement (38). However, an experiment in which people affected by writer's cramp dystonia had to perform movements with the right hand showed a gradual increase of the neural activity of the left primary sensorimotor and premotor cortices, left thalamus, and greater activation of the right cerebellum. This occurrence suggests a cerebro-cerebellar motor circuit that becomes progressively more dysfunctional with the duration of the writing motion. Because the lateral and anterior parts of the cerebellum presented an excessive activation, the function of the pathway was demonstrated. This activation is probably consecutive to the vast input that they receive from the cerebral cortex via midline crossing projections from the pontine nuclei. In contrast, the control group showed no correlation between the intensification of the activity and the duration of writing (37).

The functional connectivity within the basal ganglia-thalamo-cortical loop seems to be affected in focal hand dystonia. Altered premotor cortex (responsible for movement planning) input to basal ganglia (responsible for the selection of motor programs) is triggered by the failure of cortical-subcortical networks to maintain a balance between excitation and inhibition (39). The excess of movement that characterizes focal dystonia implies long bursts of EMG activity with co-contraction of agonist and antagonist muscles and defective fine motor control (40). This effect might occur due to the failure of several structures, such as the motor cortex, brainstem, and spinal cord, to exert proper inhibition of the movements. A decreased capacity of the globus pallidus to inhibit the thalamus can subsequently hyperactivate the medial and prefrontal cortical regions and decrease the activity in the primary motor cortex (41, 42).

Interestingly, there is an abnormal intracortical inhibition in both hemispheres, even though the manifestations are unilateral. Different types of motor cortical inhibition are caused by different inhibitory circuits, resulting in a decreased reactivity of the motor neuron during voluntary contraction (43). The basal ganglia circuits present deviating oscillatory activity and plasticity in dystonic humans. An abnormal sensorimotor integration induces the consolidation of abnormal motor engrams, which are difficult to suppress (44). However, it must be admitted that the oscillations found in the local field potentials could objectify not only volume conduction coming from remote sources, but also movement-related artifacts occurred in dystonia. Besides, evidence indicates a semi-oscillatory activity over 3–12 HZ in globus pallidus interna (GPi), which is specific to this region (45). Since the high-frequency deep brain stimulation of the GPi was proven to be an effective treatment for generalized dystonia and spasmodic torticollis (46), a possible functional link between the neural synchronization in this band and dystonia has emerged. However, additional research must be done to determine the source of the attenuation of the dystonic symptoms. It might be explained by excessive neural activity in this range of frequency or simply by the direct control of the motor cortex to the muscles (45). Also, it was suggested that too much synchrony between basal ganglia, thalamus, and cortex could impair the adequate spatiotemporal organization and sequencing of sensory input and motor output. What is more, an abnormal oscillatory activity combined with excessive synchrony might facilitate aberrant synapses in the basal ganglia and cortex (44, 47).

Furthermore, diffusion tensor imaging revealed disruptions in the white matter architecture of the motor circuit and basal ganglia in patients with idiopathic dystonia, including ones concerning axonal coherence and integrity. Mean diffusivity was increased in the white matter neighboring the pallidum, putamen, caudate, and other subcortical hemispheric regions. Since it represents a proof of a low number of cells in a big extracellular space, it is believed that dystonic individuals can present an essential loss of cells and impaired connectivity within the white matter of the previously mentioned structures. Moreover, this suggests that dystonic patients own damaged cortico-subcortical connectivity, which might cause the deficient planning and regulation of movement that they encounter. Also, the abnormalities can be associated with both focal and generalized dystonia, disregarding the genotype (48). In another study, both asymptomatic and symptomatic patients carrying the DYT1 mutation showed white matter abnormalities in the sensorimotor cortex. Nevertheless, it was impossible to demonstrate if the abnormalities were specific to the genotype represented just a piece of the pathophysiological spectrum of the disease. The gene carriers showed abnormal anatomical connectivity of the supplementary motor area, which might have allowed them to develop clinical dystonia symptoms (49).

Overall, the clinical manifestations in dystonia occur because of the described loss of inhibition, the existence of sensory dysfunction, and maladaptive synaptic plasticity. The reorganization of the central nervous system could have opposite effects on the primary motor deficit. Since several brain regions participate in movement regulation, it becomes challenging to decide whether dystonia is caused by a single neural intersection dysfunction, or it implies the participation of multiple local dysfunctional networks. It might also be caused by damaged connectivity between network points. Nevertheless, when one hemisphere is perturbed or desynchronized, the remaining tries to compensate for the deficit by increasing its baseline oscillatory cortical activity. This phenomenon will finally enhance the coordination and coherence between the two and, therefore, improve the motor competence (50). In conclusion, our study shows that local dysfunctions in the cerebellar hemispheres could induce lateralized dystonia as well as alterations in the interhemispheric motor cortex coherence. We propose that this desynchronization represents a mechanism of compensation of abnormal lateralized motor cortex oscillations.

## Data Availability Statement

The original contributions presented in the study are included in the article/Supplementary Materials, further inquiries can be directed to the corresponding author/s.

## Ethics Statement

The animal study was reviewed and approved by Ethical Committee for Animal Research of Carol Davila University of Medicine and Pharmacy, Bucharest, Romania (#PO-35-F-03).

## Author Contributions

EG, IG, CZ, LZ, DP, and A-MZ designed the experiments and wrote the manuscript. EG, IG, CZ, S-AT, and AŞ performed the experiments and analyzed the data. All authors contributed to the article and approved the submitted version.

## Conflict of Interest

The authors declare that the research was conducted in the absence of any commercial or financial relationships that could be considered a potential conflict of interest.

## Supplementary Material

Supplementary Figure 1 EMG power spectral density distribution for dystonic and unaffected sides for frequency bands D3 (125–250 Hz) **(A,B)**, band D4 (62.5–125 Hz) **(C,D)**, D5 (31.5-62 Hz) **(E,F)**.

Supplementary Figure 2 EMG recordings during movement of the mice in the arena, recordings from left and right neck muscles, post-kainate administration (duration 15 s, simultaneously with the video).

Supplementary Figure 3 EMG recordings during movement of the mice in the arena, recordings from agonist and antagonist left hindlimb muscles, post-kainate administration (duration 5 s).

Supplementary Table 1 Behavior (AW%). Dystonia score. Statistical data.

Supplementary Table 2 Electromyography parameters. Statistical data.

Supplementary Table 3 Left EMG power spectral density bands. Statistical data.

Supplementary Table 4 Right motor cortex power spectral density bands. Statistical data.

Supplementary Table 5 Right motor cortex ECoG-left EMG coherence spectral density bands. Statistical data.

Supplementary Table 6 Right EMG power spectral density bands. Statistical data.

Supplementary Table 7 Left motor cortex ECoG power spectral density bands. Statistical data.

Supplementary Table 8 Left motor cortex ECoG-right EMG coherence spectral density bands. Statistical data.

Supplementary Table 9 Left-right primary motor cortices coherence. Statistical data.

Supplementary Video 1 Video recording during movement of the mouse in the arena, post-kainate administration (duration 15 s).

Supplementary Video 2 Video recording during movement of the mouse in the arena, pre-kainate administration (duration 19 s).
