# Supplementary material for: Reduced Interhemispheric Coherence in Cerebellar Kainic Acid-Induced Lateralized Dystonia
Source: Front Neurol. 2020 Nov 23;11:580540. doi: 10.3389/fneur.2020.580540 (PMC7719699; doi:10.3389/fneur.2020.580540)
Supplement: Supplementary Table 1 — Behavior (AW%). Dystonia score. Statistical data. [file Table_1.docx]

**Supplementary Material**

***Reduced Interhemispheric Coherence in Cerebellar Kainic Acid-Induced Lateralized Dystonia***

Elena Laura Georgescu Margarint^1#^, Ioana Antoaneta Georgescu^1#^, Carmen Denise Mihaela Zahiu^1^, Stefan-Alexandru Tirlea^1^, Alexandru Răzvan Șteopoaie^1^, Leon Zăgrean^1^, Daniela Popa^1,2*^, Ana-Maria Zăgrean^1*^

^1^Division of Physiology and Neuroscience, Carol Davila University of Medicine and Pharmacy, Bucharest, Romania

^2^Institut de biologie de l'Ecole normale supérieure (IBENS), Ecole normale supérieure, CNRS, INSERM, PSL Research University, 75005 Paris, France

^#^ These authors contributed equally to this work.

*These authors jointly directed this work.

***Correspondence should be addressed to DP (**[**dpopa@bio.ens.psl.eu**](mailto:dpopa@bio.ens.psl.eu)**) or AMZ (**[**ana-maria.zagrean@umfcd.ro**](mailto:ana-maria.zagrean@umfcd.ro)**)**

**Supplementary Tables:**

Table 1. Behavior (AW%). Dystonia score. Statistical data.

| TABLE 1 \| Behavior (Figure 2) | | |
| --- | --- | --- |
| Active wake % | Test | P value |
| Pre-kainate vs Post-kainate (all days) (A) | Mann Whitney test | P= 0.0303 |
| Pre-kainate active wake (B) | Kruskall-Wallis test | P = 0.9524 |
| baseline vs. day 1 | +Dunn's multiple comparisons test | ns |
| baseline vs. day 2 | +Dunn's multiple comparisons test | ns |
| baseline vs. day 3 | +Dunn's multiple comparisons test | ns |
| baseline vs. day 4 | +Dunn's multiple comparisons test | ns |
| baseline vs. day 5 | +Dunn's multiple comparisons test | ns |
| Post-kainate active wake | Kruskall-Wallis test | P = 0.6660 |
| baseline vs. day 1 | +Dunn's multiple comparisons test | ns |
| baseline vs. day 2 | +Dunn's multiple comparisons test | ns |
| baseline vs. day 3 | +Dunn's multiple comparisons test | ns |
| baseline vs. day 4 | +Dunn's multiple comparisons test | ns |
| baseline vs. day 5 | +Dunn's multiple comparisons test | ns |
| Active wake vs. Quiet wake comparison | Multiple t-tests | ns |
| Baseline |  | P > 0.9999 |
| day 1 |  | P = 0.4577 |
| day 2 |  | P = 0.2897 |
| day 3 |  | P = 0.8306 |
| day 4 |  | P = 0.3937 |
| day 5 |  | P = 0.3867 |
| Dystonia scores (Figure 2) (C) |  |  |
| Average score per day | Friedman test | P = 0.0455 |
| day 1 vs. day 2 | +Dunn's multiple comparison test | ns |
| day 1 vs. day 3 | +Dunn's multiple comparison test | ns |
| day 1 vs. day 4 | +Dunn's multiple comparison test | ns |
| day 1 vs. day 5 | +Dunn's multiple comparison test | * |
| Dystonia scores per 30-min (D) |  |  |
|  | Two-way ANOVA | P < 0.0001 |
| 0-30 min vs. 30-60 min | +Tukey's multiple comparisons test | ns |
| 0-30 min vs. 60-90 min | +Tukey's multiple comparisons test | **** |
| 30-60 min vs. 60-90 min | +Tukey's multiple comparisons test | ** |
| Control Active wake % |  |  |
| Pre-saline vs Post-saline (all days) (E) | Mann Whitney test | P= 0.9307 |
| Hemidystonia score (G) |  |  |
| Pre-kainate vs Post-kainate (all days) | Mann Whitney test | P= 0.0079 |

Table 2. Electromyography parameters. Statistical data.

| TABLE 2 \| Electromyography (Figure 3) | | |
| --- | --- | --- |
|  | Test | P value |
| Mean EMG frequency: | | |
| Affected side (left) vs. unaffected side (right) EMG pre-kainate (B) | | |
| Mean frequency pre-kainate (left) | Kruskall-Wallis test | P=0.8669 |
| baseline vs. day 1 | +Dunn's multiple comparisons test | ns |
| baseline vs. day 2 | +Dunn's multiple comparisons test | ns |
| baseline vs. day 3 | +Dunn's multiple comparisons test | ns |
| baseline vs. day 4 | +Dunn's multiple comparisons test | ns |
| baseline vs. day 5 | +Dunn's multiple comparisons test | ns |
| Mean frequency pre-kainate (right) | Kruskall-Wallis test | P=0.1805 |
| baseline vs. day 1 | +Dunn's multiple comparisons test | ns |
| baseline vs. day 2 | +Dunn's multiple comparisons test | ns |
| baseline vs. day 3 | +Dunn's multiple comparisons test | ns |
| baseline vs. day 4 | +Dunn's multiple comparisons test | ns |
| baseline vs. day 5 | +Dunn's multiple comparisons test | ns |
| Affected side (left) vs. unaffected side (right) EMG post-kainate (C) | | |
| Mean frequency post-kainate (left) | Kruskall-Wallis test | P < 0.0001 |
| baseline vs. day 1 | +Dunn's multiple comparisons test | ns |
| baseline vs. day 2 | +Dunn's multiple comparisons test | ns |
| baseline vs. day 3 | +Dunn's multiple comparisons test | ns |
| baseline vs. day 4 | +Dunn's multiple comparisons test | ns |
| baseline vs. day 5 | +Dunn's multiple comparisons test | ns |
| Mean frequency post-kainate (right) | Kruskall-Wallis test | P= 0.0696 |
| baseline vs. day 1 | +Dunn's multiple comparisons test | ns |
| baseline vs. day 2 | +Dunn's multiple comparisons test | ns |
| baseline vs. day 3 | +Dunn's multiple comparisons test | ** |
| baseline vs. day 4 | +Dunn's multiple comparisons test | ns |
| baseline vs. day 5 | +Dunn's multiple comparisons test | ns |
| Average rectified value EMG: | | |
| Affected side (left) vs. unaffected side (right) ARV EMG post-kainate (D) | | |
| Affected side (left) | Two-way ANOVA | P < 0.0001 |
| baseline vs. day1 | +Dunnett's multiple comparisons test | ns |
| baseline vs. day2 | +Dunnett's multiple comparisons test | ns |
| baseline vs. day3 | +Dunnett's multiple comparisons test | ns |
| baseline vs. day4 | +Dunnett's multiple comparisons test | ** |
| baseline vs. day5 | +Dunnett's multiple comparisons test | ns |
| Unaffected side (right) | Two-way ANOVA | P < 0.0001 |
| baseline vs. day1 | +Dunnett's multiple comparisons test | ns |
| baseline vs. day2 | +Dunnett's multiple comparisons test | ns |
| baseline vs. day3 | +Dunnett's multiple comparisons test | * |
| baseline vs. day4 | +Dunnett's multiple comparisons test | ns |
| baseline vs. day5 | +Dunnett's multiple comparisons test | ns |
| Affected (left) side EMG (E) | | |
| Pre-kainate | Two-way ANOVA | P = 0.0017 |
| baseline vs. day1 | +Dunnett's multiple comparisons test | ns |
| baseline vs. day2 | +Dunnett's multiple comparisons test | ns |
| baseline vs. day3 | +Dunnett's multiple comparisons test | ns |
| baseline vs. day4 | +Dunnett's multiple comparisons test | ns |
| baseline vs. day5 | +Dunnett's multiple comparisons test | ns |
| Post-kainate | Two-way ANOVA | P = 0.0017 |
| baseline vs. day1 | +Dunnett's multiple comparisons test | ns |
| baseline vs. day2 | +Dunnett's multiple comparisons test | ns |
| baseline vs. day3 | +Dunnett's multiple comparisons test | ns |
| baseline vs. day4 | +Dunnett's multiple comparisons test | * |
| baseline vs. day5 | +Dunnett's multiple comparisons test | ns |
| Unaffected side (right) EMG (H) | | |
| Pre-kainate | Two-way ANOVA | P < 0.0001 |
| baseline vs. day1 | +Dunnett's multiple comparisons test | ns |
| baseline vs. day2 | +Dunnett's multiple comparisons test | ns |
| baseline vs. day3 | +Dunnett's multiple comparisons test | ** |
| baseline vs. day4 | +Dunnett's multiple comparisons test | ** |
| baseline vs. day5 | +Dunnett's multiple comparisons test | ns |
| Post-kainate | Two-way ANOVA | P < 0.0001 |
| baseline vs. day1 | +Dunnett's multiple comparisons test | ns |
| baseline vs. day2 | +Dunnett's multiple comparisons test | ns |
| baseline vs. day3 | +Dunnett's multiple comparisons test | * |
| baseline vs. day4 | +Dunnett's multiple comparisons test | * |
| baseline vs. day5 | +Dunnett's multiple comparisons test | ns |
| Root mean square EMG: | | |
| Affected side (left) EMG (F) | |  |
| Pre-kainate | Two-way ANOVA | P = 0.7436 |
| baseline vs. day1 | +Dunnett's multiple comparisons test | ns |
| baseline vs. day2 | +Dunnett's multiple comparisons test | ns |
| baseline vs. day3 | +Dunnett's multiple comparisons test | ns |
| baseline vs. day4 | +Dunnett's multiple comparisons test | ns |
| baseline vs. day5 | +Dunnett's multiple comparisons test | ns |
| Post-kainate | Two-way ANOVA | P = 0.7436 |
| baseline vs. day1 | +Dunnett's multiple comparisons test | ns |
| baseline vs. day2 | +Dunnett's multiple comparisons test | ns |
| baseline vs. day3 | +Dunnett's multiple comparisons test | ns |
| baseline vs. day4 | +Dunnett's multiple comparisons test | ns |
| baseline vs. day5 | +Dunnett's multiple comparisons test | ns |
| Unaffected side (right) EMG (I) | | |
| Pre-kainate | Two-way ANOVA | P = 0.4784 |
| baseline vs. day1 | +Dunnett's multiple comparisons test | ns |
| baseline vs. day2 | +Dunnett's multiple comparisons test | ns |
| baseline vs. day3 | +Dunnett's multiple comparisons test | ns |
| baseline vs. day4 | +Dunnett's multiple comparisons test | ns |
| baseline vs. day5 | +Dunnett's multiple comparisons test | ns |
| Post-kainate | Two-way ANOVA | P = 0.4784 |
| baseline vs. day1 | +Dunnett's multiple comparisons test | ns |
| baseline vs. day2 | +Dunnett's multiple comparisons test | ns |
| baseline vs. day3 | +Dunnett's multiple comparisons test | ns |
| baseline vs. day4 | +Dunnett's multiple comparisons test | ns |
| baseline vs. day5 | +Dunnett's multiple comparisons test | ns |
| Affected side (left) vs. unaffected side (right) RMS EMG post-kainate (G) | | |
| Affected side (left) | Two-way ANOVA | P = 0.2432 |
| baseline vs. day1 | +Dunnett's multiple comparisons test | ns |
| baseline vs. day2 | +Dunnett's multiple comparisons test | ns |
| baseline vs. day3 | +Dunnett's multiple comparisons test | ns |
| baseline vs. day4 | +Dunnett's multiple comparisons test | ns |
| baseline vs. day5 | +Dunnett's multiple comparisons test | ns |
| Unaffected side (right) | Two-way ANOVA | P = 0.2432 |
| baseline vs. day1 | +Dunnett's multiple comparisons test | ns |
| baseline vs. day2 | +Dunnett's multiple comparisons test | ns |
| baseline vs. day3 | +Dunnett's multiple comparisons test | ns |
| baseline vs. day4 | +Dunnett's multiple comparisons test | ns |
| baseline vs. day5 | +Dunnett's multiple comparisons test | ns |

Table 3. Left EMG power spectral density bands. Statistical data.

| TABLE 3 \| Power spectral density of the left EMG (Figure 4 III) | | |
| --- | --- | --- |
| Affected side EMG | Test | P value |
| Delta pre-kainate (A) | Kruskal-Wallis test | P = 0.0006 |
| Baseline vs. day 1 | +Dunn's multiple comparison test | ns |
| Baseline vs. day 2 | +Dunn's multiple comparison test | ns |
| Baseline vs. day 3 | +Dunn's multiple comparison test | * |
| Baseline vs. day 4 | +Dunn's multiple comparison test | ** |
| Baseline vs. day 5 | +Dunn's multiple comparison test | ** |
| Delta post-kainate | Kruskal-Wallis test | P = 0.0003 |
| Baseline vs. day 1 | +Dunn's multiple comparison test | ns |
| Baseline vs. day 2 | +Dunn's multiple comparison test | ns |
| Baseline vs. day 3 | +Dunn's multiple comparison test | ns |
| Baseline vs. day 4 | +Dunn's multiple comparison test | ** |
| Baseline vs. day 5 | +Dunn's multiple comparison test | ns |
| Theta pre-kainate (B) | Kruskal-Wallis test | P = 0.0003 |
| Baseline vs. day 1 | +Dunn's multiple comparison test | ns |
| Baseline vs. day 2 | +Dunn's multiple comparison test | ns |
| Baseline vs. day 3 | +Dunn's multiple comparison test | * |
| Baseline vs. day 4 | +Dunn's multiple comparison test | ** |
| Baseline vs. day 5 | +Dunn's multiple comparison test | ** |
| Theta post-kainate | Kruskal-Wallis test | P = 0.0002 |
| Baseline vs. day 1 | +Dunn's multiple comparison test | ns |
| Baseline vs. day 2 | +Dunn's multiple comparison test | ns |
| Baseline vs. day 3 | +Dunn's multiple comparison test | * |
| Baseline vs. day 4 | +Dunn's multiple comparison test | ** |
| Baseline vs. day 5 | +Dunn's multiple comparison test | ns |
| Beta pre-kainate (C) | Kruskal-Wallis test | P = 0.0001 |
| Baseline vs. day 1 | +Dunn's multiple comparison test | ns |
| Baseline vs. day 2 | +Dunn's multiple comparison test | ns |
| Baseline vs. day 3 | +Dunn's multiple comparison test | ** |
| Baseline vs. day 4 | +Dunn's multiple comparison test | *** |
| Baseline vs. day 5 | +Dunn's multiple comparison test | ** |
| Beta post-kainate | Kruskal-Wallis test | P = 0.0008 |
| Baseline vs. day 1 | Dunn's multiple comparisons test | ns |
| Baseline vs. day 2 | Dunn's multiple comparisons test | ns |
| Baseline vs. day 3 | Dunn's multiple comparisons test | * |
| Baseline vs. day 4 | Dunn's multiple comparisons test | ** |
| Baseline vs. day 5 | Dunn's multiple comparisons test | ns |
| Low gamma pre-kainate (D) | Kruskal-Wallis test | P = 0.0003 |
| Baseline vs. day 1 | +Dunn's multiple comparison test | ns |
| Baseline vs. day 2 | +Dunn's multiple comparison test | ns |
| Baseline vs. day 3 | +Dunn's multiple comparison test | ** |
| Baseline vs. day 4 | +Dunn's multiple comparison test | ** |
| Baseline vs. day 5 | +Dunn's multiple comparison test | ** |
| Low gamma post-kainate | Kruskal-Wallis test | P = 0.0008 |
| Baseline vs. day 1 | +Dunn's multiple comparison test | ns |
| Baseline vs. day 2 | +Dunn's multiple comparison test | ns |
| Baseline vs. day 3 | +Dunn's multiple comparison test | ns |
| Baseline vs. day 4 | +Dunn's multiple comparison test | ** |
| Baseline vs. day 5 | +Dunn's multiple comparison test | * |
| High gamma pre-kainate (E) | Kruskal-Wallis test | P = 0.0091 |
| Baseline vs. day 1 | +Dunn's multiple comparison test | ns |
| Baseline vs. day 2 | +Dunn's multiple comparison test | ns |
| Baseline vs. day 3 | +Dunn's multiple comparison test | ns |
| Baseline vs. day 4 | +Dunn's multiple comparison test | * |
| Baseline vs. day 5 | +Dunn's multiple comparison test | * |
| High gamma post-kainate | Kruskal-Wallis test | P = 0.0102 |
| Baseline vs. day 1 | +Dunn's multiple comparison test | ns |
| Baseline vs. day 2 | +Dunn's multiple comparison test | ns |
| Baseline vs. day 3 | +Dunn's multiple comparison test | ns |
| Baseline vs. day 4 | +Dunn's multiple comparison test | ns |
| Baseline vs. day 5 | +Dunn's multiple comparison test | * |
| Delta pre-kainate (A) |  |  |
| day 3 vs. day 4 | Wilcoxon matched-pairs signed rank test | P = 0.7422 |
| day 4 vs. day 5 | Wilcoxon matched-pairs signed rank test | P = 0.9375 |
| Delta post-kainate |  |  |
| day 3 vs. day 4 | Wilcoxon matched-pairs signed rank test | P = 0.2405 |
| day 4 vs. day 5 | Wilcoxon matched-pairs signed rank test | P = 0.1434 |
| Theta pre-kainate (B) |  |  |
| day 3 vs. day 4 | Wilcoxon matched-pairs signed rank test | P = 0.7422 |
| day 4 vs. day 5 | Wilcoxon matched-pairs signed rank test | P = 0.5781 |
| Theta post-kainate |  |  |
| day 3 vs. day 4 | Wilcoxon matched-pairs signed rank test | P = 0.4908 |
| day 4 vs. day 5 | Wilcoxon matched-pairs signed rank test | P = 0.1515 |
| Beta pre-kainate (C) |  |  |
| day 3 vs. day 4 | Wilcoxon matched-pairs signed rank test | P = 0.6406 |
| day 4 vs. day 5 | Wilcoxon matched-pairs signed rank test | P = 0.4688 |
| Beta post-kainate |  |  |
| day 3 vs. day 4 | Mann Whitney test | P = 0.4655 |
| day 4 vs. day 5 | Mann Whitney test | P = 0.3328 |
| Low gamma pre-kainate (D) |  |  |
| day 3 vs. day 4 | Wilcoxon matched-pairs signed rank test | P = 0.4609 |
| day 4 vs. day 5 | Wilcoxon matched-pairs signed rank test | P = 0.8125 |
| Low gamma post-kainate |  |  |
| day 3 vs. day 4 | Wilcoxon matched-pairs signed rank test | P = 0.2405 |
| day 4 vs. day 5 | Wilcoxon matched-pairs signed rank test | P = 0.684 |
| High gamma pre-kainate (E) |  |  |
| day 3 vs. day 4 | Wilcoxon matched-pairs signed rank test | P = 0.7422 |
| day 4 vs. day 5 | Wilcoxon matched-pairs signed rank test | P > 0.9999 |
| High gamma post-kainate |  |  |
| day 3 vs. day 4 | Wilcoxon matched-pairs signed rank test | P = 0.5088 |
| day 4 vs. day 5 | Wilcoxon matched-pairs signed rank test | P = 0.4389 |
| D3 band EMG affected side  Post-kainate vs. Pre-kainate | Wilcoxon matched-pairs signed rank test | P = 0.0625 |
| D4 band EMG affected side  Post-kainate vs. Pre-kainate | Wilcoxon matched-pairs signed rank test | P = 0.0625 |
| D5 band EMG affected side  Post-kainate vs. Pre-kainate | Wilcoxon matched-pairs signed rank test | P = 0.0625 |

Table 4. Right motor cortex power spectral density bands. Statistical data.

| TABLE 4 \| Right motor cortex ECoG power spectral density (Figure 4 I) | | |
| --- | --- | --- |
| Affected side | Test | P value |
| Delta pre-kainate (A) | Kruskal-Wallis test | P = 0.0009 |
| Baseline vs. day 1 | +Dunn's multiple comparison test | ns |
| Baseline vs. day 2 | +Dunn's multiple comparison test | ns |
| Baseline vs. day 3 | +Dunn's multiple comparison test | ns |
| Baseline vs. day 4 | +Dunn's multiple comparison test | ns |
| Baseline vs. day 5 | +Dunn's multiple comparison test | *** |
| Delta post-kainate | Kruskal-Wallis test | P = 0.0024 |
| Baseline vs. day 1 | +Dunn's multiple comparison test | ns |
| Baseline vs. day 2 | +Dunn's multiple comparison test | ns |
| Baseline vs. day 3 | +Dunn's multiple comparison test | ns |
| Baseline vs. day 4 | +Dunn's multiple comparison test | ns |
| Baseline vs. day 5 | +Dunn's multiple comparison test | ns |
| Theta pre-kainate (B) | Kruskal-Wallis test | P = 0.4052 |
| Baseline vs. day 1 | +Dunn's multiple comparison test | ns |
| Baseline vs. day 2 | +Dunn's multiple comparison test | ns |
| Baseline vs. day 3 | +Dunn's multiple comparison test | ns |
| Baseline vs. day 4 | +Dunn's multiple comparison test | ns |
| Baseline vs. day 5 | +Dunn's multiple comparison test | ns |
| Theta post-kainate | Kruskal-Wallis test | P = 0.0227 |
| Baseline vs. day 1 | +Dunn's multiple comparison test | ** |
| Baseline vs. day 2 | +Dunn's multiple comparison test | ns |
| Baseline vs. day 3 | +Dunn's multiple comparison test | ns |
| Baseline vs. day 4 | +Dunn's multiple comparison test | ns |
| Baseline vs. day 5 | +Dunn's multiple comparison test | ns |
| Beta pre-kainate (C) | Kruskal-Wallis test | P = 0.2660 |
| Baseline vs. day 1 | +Dunn's multiple comparison test | ns |
| Baseline vs. day 2 | +Dunn's multiple comparison test | ns |
| Baseline vs. day 3 | +Dunn's multiple comparison test | ns |
| Baseline vs. day 4 | +Dunn's multiple comparison test | ns |
| Baseline vs. day 5 | +Dunn's multiple comparison test | ns |
| Beta post-kainate | Kruskal-Wallis test | P = 0.0376 |
| Baseline vs. day 1 | +Dunn's multiple comparison test | * |
| Baseline vs. day 2 | +Dunn's multiple comparison test | ns |
| Baseline vs. day 3 | +Dunn's multiple comparison test | ns |
| Baseline vs. day 4 | +Dunn's multiple comparison test | ns |
| Baseline vs. day 5 | +Dunn's multiple comparison test | ns |
| Low gamma pre-kainate (D) | Kruskal-Wallis test | P = 0.0172 |
| Baseline vs. day 1 | +Dunn's multiple comparison test | ns |
| Baseline vs. day 2 | +Dunn's multiple comparison test | ns |
| Baseline vs. day 3 | +Dunn's multiple comparison test | ns |
| Baseline vs. day 4 | +Dunn's multiple comparison test | ns |
| Baseline vs. day 5 | +Dunn's multiple comparison test | * |
| Low gamma post-kainate | Kruskal-Wallis test | P = 0.3644 |
| Baseline vs. day 1 | +Dunn's multiple comparison test | ns |
| Baseline vs. day 2 | +Dunn's multiple comparison test | ns |
| Baseline vs. day 3 | +Dunn's multiple comparison test | ns |
| Baseline vs. day 4 | +Dunn's multiple comparison test | ns |
| Baseline vs. day 5 | +Dunn's multiple comparison test | ns |
| High gamma pre-kainate (E) | Kruskal-Wallis test | P = 0.0068 |
| Baseline vs. day 1 | +Dunn's multiple comparison test | ns |
| Baseline vs. day 2 | +Dunn's multiple comparison test | ns |
| Baseline vs. day 3 | +Dunn's multiple comparison test | ns |
| Baseline vs. day 4 | +Dunn's multiple comparison test | * |
| Baseline vs. day 5 | +Dunn's multiple comparison test | * |
| High gamma post-kainate | Kruskal-Wallis test | P = 0.3814 |
| Baseline vs. day 1 | +Dunn's multiple comparison test | ns |
| Baseline vs. day 2 | +Dunn's multiple comparison test | ns |
| Baseline vs. day 3 | +Dunn's multiple comparison test | ns |
| Baseline vs. day 4 | +Dunn's multiple comparison test | ns |
| Baseline vs. day 5 | +Dunn's multiple comparison test | ns |
| Delta pre-kainate (A) |  |  |
| day 3 vs. day 4 | Wilcoxon matched-pairs signed rank test | P = 0.5703 |
| day 4 vs. day 5 | Wilcoxon matched-pairs signed rank test | P = 0.0391 |
| Delta post-kainate |  |  |
| day 3 vs. day 4 | Wilcoxon matched-pairs signed rank test | P = 0.0646 |
| day 4 vs. day 5 | Wilcoxon matched-pairs signed rank test | P = 0.1688 |
| Theta pre-kainate (B) |  |  |
| day 3 vs. day 4 | Wilcoxon matched-pairs signed rank test | P = 0.4258 |
| day 4 vs. day 5 | Wilcoxon matched-pairs signed rank test | P = 0.0781 |
| Theta post-kainate |  |  |
| day 3 vs. day 4 | Wilcoxon matched-pairs signed rank test | P = 0.1688 |
| day 4 vs. day 5 | Wilcoxon matched-pairs signed rank test | P = 0.2182 |
| Beta pre-kainate (C) |  |  |
| day 3 vs. day 4 | Wilcoxon matched-pairs signed rank test | P = 0.6523 |
| day 4 vs. day 5 | Wilcoxon matched-pairs signed rank test | P = 0.0391 |
| Beta post-kainate |  |  |
| day 3 vs. day 4 | Mann Whitney test | P = 0.958 |
| day 4 vs. day 5 | Mann Whitney test | P = 0.1073 |
| Low gamma pre-kainate (D) |  |  |
| day 3 vs. day 4 | Wilcoxon matched-pairs signed rank test | P = 0.4258 |
| day 4 vs. day 5 | Wilcoxon matched-pairs signed rank test | P = 0.25 |
| Low gamma post-kainate |  |  |
| day 3 vs. day 4 | Wilcoxon matched-pairs signed rank test | P = 0.7898 |
| day 4 vs. day 5 | Wilcoxon matched-pairs signed rank test | P = 0.3902 |
| High gamma pre-kainate (E) |  |  |
| day 3 vs. day 4 | Wilcoxon matched-pairs signed rank test | P = 0.2031 |
| day 4 vs. day 5 | Wilcoxon matched-pairs signed rank test | P = 0.3828 |
| High gamma post-kainate |  |  |
| day 3 vs. day 4 | Wilcoxon matched-pairs signed rank test | P = 0.2292 |
| day 4 vs. day 5 | Wilcoxon matched-pairs signed rank test | P = 0.8996 |

Table 5. Right motor cortex ECoG-left EMG coherence spectral density bands. Statistical data.

| TABLE 5 \| EcoG - EMG coherence (affected side) (Figure 5 I) | | | |
| --- | --- | --- | --- |
| Right EcoG – left EMG | Test | F_DFn, DFd_ | P value |
| Delta pre-kainate (A) | Kruskal-Wallis |  | P = 0.6884 |
| Baseline vs. day 1 | +Dunn's multiple comparison test |  | ns |
| Baseline vs. day 2 | +Dunn's multiple comparison test |  | ns |
| Baseline vs. day 3 | +Dunn's multiple comparison test |  | ns |
| Baseline vs. day 4 | +Dunn's multiple comparison test |  | ns |
| Baseline vs. day 5 | +Dunn's multiple comparison test |  | ns |
| Delta post-kainate | Kruskal-Wallis |  | P = 0.0264 |
| Baseline vs. day 1 | +Dunn's multiple comparison test |  | ns |
| Baseline vs. day 2 | +Dunn's multiple comparison test |  | ns |
| Baseline vs. day 3 | +Dunn's multiple comparison test |  | ns |
| Baseline vs. day 4 | +Dunn's multiple comparison test |  | ns |
| Baseline vs. day 5 | +Dunn's multiple comparison test |  | ns |
| Theta pre-kainate (B) | Kruskal-Wallis |  | P = 0.3863 |
| Baseline vs. day 1 | +Dunn's multiple comparison test |  | ns |
| Baseline vs. day 2 | +Dunn's multiple comparison test |  | ns |
| Baseline vs. day 3 | +Dunn's multiple comparison test |  | ns |
| Baseline vs. day 4 | +Dunn's multiple comparison test |  | ns |
| Baseline vs. day 5 | +Dunn's multiple comparison test |  | ns |
| Theta post-kainate | Kruskal-Wallis |  | P = 0.0025 |
| Baseline vs. day 1 | +Dunn's multiple comparison test |  | ns |
| Baseline vs. day 2 | +Dunn's multiple comparison test |  | ns |
| Baseline vs. day 3 | +Dunn's multiple comparison test |  | ns |
| Baseline vs. day 4 | +Dunn's multiple comparison test |  | ns |
| Baseline vs. day 5 | +Dunn's multiple comparison test |  | ns |
| Beta pre-kainate (C) | Kruskal-Wallis |  | P = 0.8787 |
| Baseline vs. day 1 | +Dunn's multiple comparison test |  | ns |
| Baseline vs. day 2 | +Dunn's multiple comparison test |  | ns |
| Baseline vs. day 3 | +Dunn's multiple comparison test |  | ns |
| Baseline vs. day 4 | +Dunn's multiple comparison test |  | ns |
| Baseline vs. day 5 | +Dunn's multiple comparison test |  | ns |
| Beta post-kainate | 1-way ANOVA | F (5, 138) = 1.139 | P = 0.3430 |
| Baseline vs. day 1 | +Dunnett's multiple comparisons test |  | ns |
| Baseline vs. day 2 | +Dunnett's multiple comparisons test |  | ns |
| Baseline vs. day 3 | +Dunnett's multiple comparisons test |  | ns |
| Baseline vs. day 4 | +Dunnett's multiple comparisons test |  | ns |
| Baseline vs. day 5 | +Dunnett's multiple comparisons test |  | ns |
| Low gamma pre-kainate (D) | Kruskal-Wallis |  | P = 0.0713 |
| Baseline vs. day 1 | +Dunn's multiple comparison test |  | ns |
| Baseline vs. day 2 | +Dunn's multiple comparison test |  | ns |
| Baseline vs. day 3 | +Dunn's multiple comparison test |  | ns |
| Baseline vs. day 4 | +Dunn's multiple comparison test |  | ns |
| Baseline vs. day 5 | +Dunn's multiple comparison test |  | ns |
| Low gamma post-kainate | Kruskal-Wallis |  | P = 0.0047 |
| Baseline vs. day 1 | +Dunn's multiple comparison test |  | ns |
| Baseline vs. day 2 | +Dunn's multiple comparison test |  | ns |
| Baseline vs. day 3 | +Dunn's multiple comparison test |  | * |
| Baseline vs. day 4 | +Dunn's multiple comparison test |  | * |
| Baseline vs. day 5 | +Dunn's multiple comparison test |  | ns |
| High gamma pre-kainate (E) | Kruskal-Wallis |  | P = 0.0209 |
| Baseline vs. day 1 | +Dunn's multiple comparison test |  | ns |
| Baseline vs. day 2 | +Dunn's multiple comparison test |  | ns |
| Baseline vs. day 3 | +Dunn's multiple comparison test |  | ns |
| Baseline vs. day 4 | +Dunn's multiple comparison test |  | ns |
| Baseline vs. day 5 | +Dunn's multiple comparison test |  | * |
| High gamma post-kainate | Kruskal-Wallis |  | P = 0.0011 |
| Baseline vs. day 1 | +Dunn's multiple comparison test |  | ns |
| Baseline vs. day 2 | +Dunn's multiple comparison test |  | ns |
| Baseline vs. day 3 | +Dunn's multiple comparison test |  | * |
| Baseline vs. day 4 | +Dunn's multiple comparison test |  | * |
| Baseline vs. day 5 | +Dunn's multiple comparison test |  | ns |
| Delta pre-kainate (A) |  |  |  |
| day 3 vs. day 4 | Wilcoxon matched-pairs signed rank test |  | P = 0.6875 |
| day 4 vs. day 5 | Wilcoxon matched-pairs signed rank test |  | P = 0.9102 |
| Delta post-kainate |  |  |  |
| day 3 vs. day 4 | Wilcoxon matched-pairs signed rank test |  | P = 0.0003 |
| day 4 vs. day 5 | Wilcoxon matched-pairs signed rank test |  | P = 0.8237 |
| Theta pre-kainate (B) |  |  |  |
| day 3 vs. day 4 | Wilcoxon matched-pairs signed rank test |  | P > 0.9999 |
| day 4 vs. day 5 | Wilcoxon matched-pairs signed rank test |  | P = 0.4961 |
| Theta post-kainate |  |  |  |
| day 3 vs. day 4 | Wilcoxon matched-pairs signed rank test |  | P = 0.0007 |
| day 4 vs. day 5 | Wilcoxon matched-pairs signed rank test |  | P = 0.6102 |
| Beta pre-kainate (C) |  |  |  |
| day 3 vs. day 4 | Wilcoxon matched-pairs signed rank test |  | P = 0.8125 |
| day 4 vs. day 5 | Wilcoxon matched-pairs signed rank test |  | P = 0.2500 |
| Beta post-kainate |  |  |  |
| day 3 vs. day 4 | Mann Whitney test |  | P = 0.6409 |
| day 4 vs. day 5 | Mann Whitney test |  | P = 0.8811 |
| Low gamma pre-kainate (D) |  |  |  |
| day 3 vs. day 4 | Wilcoxon matched-pairs signed rank test |  | P = 0.9375 |
| day 4 vs. day 5 | Wilcoxon matched-pairs signed rank test |  | P > 0.9999 |
| Low gamma post-kainate |  |  |  |
| day 3 vs. day 4 | Wilcoxon matched-pairs signed rank test |  | P = 0.3880 |
| day 4 vs. day 5 | Wilcoxon matched-pairs signed rank test |  | P = 0.5661 |
| High gamma pre-kainate (E) |  |  |  |
| day 3 vs. day 4 | Wilcoxon matched-pairs signed rank test |  | P = 0.6875 |
| day 4 vs. day 5 | Wilcoxon matched-pairs signed rank test |  | P = 0.2031 |
| High gamma post-kainate |  |  |  |
| day 3 vs. day 4 | Wilcoxon matched-pairs signed rank test |  | P = 0.9746 |
| day 4 vs. day 5 | Wilcoxon matched-pairs signed rank test |  | P = 0.6327 |

Table 6. Right EMG power spectral density bands. Statistical data.

| TABLE 6 \| Power spectral density of the right EMG (Figure 4 IV) | | | |
| --- | --- | --- | --- |
| Unaffected side EMG | Test | F_DFn, DFd_ | P value |
| Delta pre-kainate (A) | Kruskal-Wallis test |  | P <0.0001 |
| Baseline vs. day 1 | +Dunn's multiple comparison test |  | ns |
| Baseline vs. day 2 | +Dunn's multiple comparison test |  | ns |
| Baseline vs. day 3 | +Dunn's multiple comparison test |  | ** |
| Baseline vs. day 4 | +Dunn's multiple comparison test |  | *** |
| Baseline vs. day 5 | +Dunn's multiple comparison test |  | * |
| Delta post-kainate | Kruskal-Wallis test |  | P <0.0001 |
| Baseline vs. day 1 | +Dunn's multiple comparison test |  | ns |
| Baseline vs. day 2 | +Dunn's multiple comparison test |  | ns |
| Baseline vs. day 3 | +Dunn's multiple comparison test |  | *** |
| Baseline vs. day 4 | +Dunn's multiple comparison test |  | ** |
| Baseline vs. day 5 | +Dunn's multiple comparison test |  | ns |
| Theta pre-kainate (B) | Kruskal-Wallis test |  | P =0.0002 |
| Baseline vs. day 1 | +Dunn's multiple comparison test |  | ns |
| Baseline vs. day 2 | +Dunn's multiple comparison test |  | ns |
| Baseline vs. day 3 | +Dunn's multiple comparison test |  | ** |
| Baseline vs. day 4 | +Dunn's multiple comparison test |  | *** |
| Baseline vs. day 5 | +Dunn's multiple comparison test |  | * |
| Theta post-kainate | Kruskal-Wallis test |  | P =0.0001 |
| Baseline vs. day 1 | +Dunn's multiple comparison test |  | ns |
| Baseline vs. day 2 | +Dunn's multiple comparison test |  | ns |
| Baseline vs. day 3 | +Dunn's multiple comparison test |  | ** |
| Baseline vs. day 4 | +Dunn's multiple comparison test |  | ** |
| Baseline vs. day 5 | +Dunn's multiple comparison test |  | ns |
| Beta pre-kainate (C) | Kruskal-Wallis test |  | P <0.0001 |
| Baseline vs. day 1 | +Dunn's multiple comparison test |  | ns |
| Baseline vs. day 2 | +Dunn's multiple comparison test |  | ns |
| Baseline vs. day 3 | +Dunn's multiple comparison test |  | ** |
| Baseline vs. day 4 | +Dunn's multiple comparison test |  | *** |
| Baseline vs. day 5 | +Dunn's multiple comparison test |  | * |
| Beta post-kainate | Kruskal-Wallis test | F(5, 153)  =5.981 | P <0.0001 |
| Baseline vs. day 1 | +Dunn's multiple comparison test |  | ns |
| Baseline vs. day 2 | +Dunn's multiple comparison test |  | ns |
| Baseline vs. day 3 | +Dunn's multiple comparison test |  | ** |
| Baseline vs. day 4 | +Dunn's multiple comparison test |  | ** |
| Baseline vs. day 5 | +Dunn's multiple comparison test |  | ns |
| Low gamma pre-kainate (D) | Kruskal-Wallis test |  | P <0.0001 |
| Baseline vs. day 1 | +Dunn's multiple comparison test |  | ns |
| Baseline vs. day 2 | +Dunn's multiple comparison test |  | * |
| Baseline vs. day 3 | +Dunn's multiple comparison test |  | ** |
| Baseline vs. day 4 | +Dunn's multiple comparison test |  | *** |
| Baseline vs. day 5 | +Dunn's multiple comparison test |  | ** |
| Low gamma post-kainate | Kruskal-Wallis test |  | P =0.0008 |
| Baseline vs. day 1 | +Dunn's multiple comparison test |  | ns |
| Baseline vs. day 2 | +Dunn's multiple comparison test |  | ns |
| Baseline vs. day 3 | +Dunn's multiple comparison test |  | * |
| Baseline vs. day 4 | +Dunn's multiple comparison test |  | * |
| Baseline vs. day 5 | +Dunn's multiple comparison test |  | * |
| High gamma pre-kainate (E) | Kruskal-Wallis test |  | P =0.0041 |
| Baseline vs. day 1 | +Dunn's multiple comparison test |  | ns |
| Baseline vs. day 2 | +Dunn's multiple comparison test |  | ns |
| Baseline vs. day 3 | +Dunn's multiple comparison test |  | * |
| Baseline vs. day 4 | +Dunn's multiple comparison test |  | * |
| Baseline vs. day 5 | +Dunn's multiple comparison test |  | * |
| High gamma post-kainate | Kruskal-Wallis test |  | P =0.0110 |
| Baseline vs. day 1 | +Dunn's multiple comparison test |  | ns |
| Baseline vs. day 2 | +Dunn's multiple comparison test |  | ns |
| Baseline vs. day 3 | +Dunn's multiple comparison test |  | ns |
| Baseline vs. day 4 | +Dunn's multiple comparison test |  | * |
| Baseline vs. day 5 | +Dunn's multiple comparison test |  | ns |
| Delta pre-kainate (A) |  |  |  |
| day 3 vs. day 4 | Wilcoxon matched-pairs signed rank test |  | P =0.9102 |
| day 4 vs. day 5 | Wilcoxon matched-pairs signed rank test |  | P >0.9999 |
| Delta post-kainate |  |  |  |
| day 3 vs. day 4 | Wilcoxon matched-pairs signed rank test |  | P =0.4245 |
| day 4 vs. day 5 | Wilcoxon matched-pairs signed rank test |  | P =0.0542 |
| Theta pre-kainate (B) |  |  |  |
| day 3 vs. day 4 | Wilcoxon matched-pairs signed rank test |  | P =0.5703 |
| day 4 vs. day 5 | Wilcoxon matched-pairs signed rank test |  | P =0.5625 |
| Theta post-kainate |  |  |  |
| day 3 vs. day 4 | Wilcoxon matched-pairs signed rank test |  | P =0.5235 |
| day 4 vs. day 5 | Wilcoxon matched-pairs signed rank test |  | P =0.0634 |
| Beta pre-kainate (C) |  |  |  |
| day 3 vs. day 4 | Wilcoxon matched-pairs signed rank test |  | P =0.9102 |
| day 4 vs. day 5 | Wilcoxon matched-pairs signed rank test |  | P =0.8438 |
| Beta post-kainate |  |  |  |
| day 3 vs. day 4 | Mann Whitney test |  | P =0.8571 |
| day 4 vs. day 5 | Mann Whitney test |  | P =0.0904 |
| Low gamma pre-kainate (D) |  |  |  |
| day 3 vs. day 4 | Wilcoxon matched-pairs signed rank test |  | P =0.9102 |
| day 4 vs. day 5 | Wilcoxon matched-pairs signed rank test |  | P =0.4375 |
| Low gamma post-kainate |  |  |  |
| day 3 vs. day 4 | Wilcoxon matched-pairs signed rank test |  | P =0.2902 |
| day 4 vs. day 5 | Wilcoxon matched-pairs signed rank test |  | P =0.8237 |
| High gamma pre-kainate (E) |  |  |  |
| day 3 vs. day 4 | Wilcoxon matched-pairs signed rank test |  | P =0.8203 |
| day 4 vs. day 5 | Wilcoxon matched-pairs signed rank test |  | P =0.5625 |
| High gamma post-kainate |  |  |  |
| day 3 vs. day 4 | Wilcoxon matched-pairs signed rank test |  | P =0.7024 |
| day 4 vs. day 5 | Wilcoxon matched-pairs signed rank test |  | P =0.9746 |
| D3 band EMG unaffected side | Wilcoxon matched-pairs signed rank test |  | P = 0.0625 |
| D4 band EMG unaffected side | Wilcoxon matched-pairs signed rank test |  | P = 0.0625 |
| D5 band EMG unaffected side  Post-kainate vs. Pre-kainate | Wilcoxon matched-pairs signed rank test |  | P = 0.1875 |

Table 7. Left motor cortex ECoG power spectral density bands. Statistical data.

| TABLE 7 \| Left motor cortex ECoG power spectral density (Figure 4 II) | | |
| --- | --- | --- |
| Unaffected side | Test | P value |
| Delta pre-kainate (A) | Kruskal-Wallis test | P = 0.0039 |
| Baseline vs. day 1 | +Dunn's multiple comparison test | ns |
| Baseline vs. day 2 | +Dunn's multiple comparison test | ns |
| Baseline vs. day 3 | +Dunn's multiple comparison test | ns |
| Baseline vs. day 4 | +Dunn's multiple comparison test | ns |
| Baseline vs. day 5 | +Dunn's multiple comparison test | ** |
| Delta post-kainate | Kruskal-Wallis test | P = 0.0296 |
| Baseline vs. day 1 | +Dunn's multiple comparison test | ns |
| Baseline vs. day 2 | +Dunn's multiple comparison test | ns |
| Baseline vs. day 3 | +Dunn's multiple comparison test | ns |
| Baseline vs. day 4 | +Dunn's multiple comparison test | ns |
| Baseline vs. day 5 | +Dunn's multiple comparison test | ns |
| Theta pre-kainate (B) | Kruskal-Wallis test | P = 0.5680 |
| Baseline vs. day 1 | +Dunn's multiple comparison test | ns |
| Baseline vs. day 2 | +Dunn's multiple comparison test | ns |
| Baseline vs. day 3 | +Dunn's multiple comparison test | ns |
| Baseline vs. day 4 | +Dunn's multiple comparison test | ns |
| Baseline vs. day 5 | +Dunn's multiple comparison test | ns |
| Theta post-kainate | Kruskal-Wallis test | P = 0.3001 |
| Baseline vs. day 1 | +Dunn's multiple comparison test | ns |
| Baseline vs. day 2 | +Dunn's multiple comparison test | ns |
| Baseline vs. day 3 | +Dunn's multiple comparison test | ns |
| Baseline vs. day 4 | +Dunn's multiple comparison test | ns |
| Baseline vs. day 5 | +Dunn's multiple comparison test | ns |
| Beta pre-kainate (C) | Kruskal-Wallis test | P = 0.2180 |
| Baseline vs. day 1 | +Dunn's multiple comparison test | ns |
| Baseline vs. day 2 | +Dunn's multiple comparison test | ns |
| Baseline vs. day 3 | +Dunn's multiple comparison test | ns |
| Baseline vs. day 4 | +Dunn's multiple comparison test | ns |
| Baseline vs. day 5 | +Dunn's multiple comparison test | ns |
| Beta post-kainate | Kruskal-Wallis test | P = 0.1163 |
| Baseline vs. day 1 | +Dunn's multiple comparison test | ns |
| Baseline vs. day 2 | +Dunn's multiple comparison test | ns |
| Baseline vs. day 3 | +Dunn's multiple comparison test | ns |
| Baseline vs. day 4 | +Dunn's multiple comparison test | ns |
| Baseline vs. day 5 | +Dunn's multiple comparison test | ns |
| Low gamma pre-kainate (D) | Kruskal-Wallis test | P = 0.0612 |
| Baseline vs. day 1 | +Dunn's multiple comparison test | ns |
| Baseline vs. day 2 | +Dunn's multiple comparison test | ns |
| Baseline vs. day 3 | +Dunn's multiple comparison test | ns |
| Baseline vs. day 4 | +Dunn's multiple comparison test | ns |
| Baseline vs. day 5 | +Dunn's multiple comparison test | ns |
| Low gamma post-kainate | Kruskal-Wallis test | P = 0.7491 |
| Baseline vs. day 1 | +Dunn's multiple comparison test | ns |
| Baseline vs. day 2 | +Dunn's multiple comparison test | ns |
| Baseline vs. day 3 | +Dunn's multiple comparison test | ns |
| Baseline vs. day 4 | +Dunn's multiple comparison test | ns |
| Baseline vs. day 5 | +Dunn's multiple comparison test | ns |
| High gamma pre-kainate (E) | Kruskal-Wallis test | P = 0.0294 |
| Baseline vs. day 1 | +Dunn's multiple comparison test | ns |
| Baseline vs. day 2 | +Dunn's multiple comparison test | ns |
| Baseline vs. day 3 | +Dunn's multiple comparison test | ns |
| Baseline vs. day 4 | +Dunn's multiple comparison test | ns |
| Baseline vs. day 5 | +Dunn's multiple comparison test | ns |
| High gamma post-kainate | Kruskal-Wallis test | P = 0.2399 |
| Baseline vs. day 1 | +Dunn's multiple comparison test | ns |
| Baseline vs. day 2 | +Dunn's multiple comparison test | ns |
| Baseline vs. day 3 | +Dunn's multiple comparison test | ns |
| Baseline vs. day 4 | +Dunn's multiple comparison test | ns |
| Baseline vs. day 5 | +Dunn's multiple comparison test | ns |
| Delta pre-kainate (A) |  |  |
| day 3 vs. day 4 | Wilcoxon matched-pairs signed rank test | P = 0.4961 |
| day 4 vs. day 5 | Wilcoxon matched-pairs signed rank test | P = 0.0547 |
| Delta post-kainate |  |  |
| day 3 vs. day 4 | Wilcoxon matched-pairs signed rank test | P = 0.4374 |
| day 4 vs. day 5 | Wilcoxon matched-pairs signed rank test | P = 0.6893 |
| Theta pre-kainate (B) |  |  |
| day 3 vs. day 4 | Wilcoxon matched-pairs signed rank test | P = 0.3008 |
| day 4 vs. day 5 | Wilcoxon matched-pairs signed rank test | P = 0.25 |
| Theta post-kainate |  |  |
| day 3 vs. day 4 | Wilcoxon matched-pairs signed rank test | P = 0.671 |
| day 4 vs. day 5 | Wilcoxon matched-pairs signed rank test | P = 0.3941 |
| Beta pre-kainate (C) |  |  |
| day 3 vs. day 4 | Wilcoxon matched-pairs signed rank test | P = 0.4258 |
| day 4 vs. day 5 | Wilcoxon matched-pairs signed rank test | P = 0.1094 |
| Beta post-kainate |  |  |
| day 3 vs. day 4 | Mann Whitney test | P = 0.6778 |
| day 4 vs. day 5 | Mann Whitney test | P = 0.2852 |
| Low gamma pre-kainate (D) |  |  |
| day 3 vs. day 4 | Wilcoxon matched-pairs signed rank test | P = 0.3008 |
| day 4 vs. day 5 | Wilcoxon matched-pairs signed rank test | P = 0.3125 |
| Low gamma post-kainate |  |  |
| day 3 vs. day 4 | Wilcoxon matched-pairs signed rank test | P = 0.8613 |
| day 4 vs. day 5 | Wilcoxon matched-pairs signed rank test | P = 0.7078 |
| High gamma pre-kainate (E) |  |  |
| day 3 vs. day 4 | Wilcoxon matched-pairs signed rank test | P = 0.25 |
| day 4 vs. day 5 | Wilcoxon matched-pairs signed rank test | P = 0.4609 |
| High gamma post-kainate |  |  |
| day 3 vs. day 4 | Wilcoxon matched-pairs signed rank test | P = 0.6171 |
| day 4 vs. day 5 | Wilcoxon matched-pairs signed rank test | P = 0.8417 |

Table 8. Left motor cortex ECoG-right EMG coherence spectral density bands. Statistical data.

| TABLE 8 \| EcoG - EMG coherence (unaffected side) (Figure 5 II) | | |
| --- | --- | --- |
| Left ECoG - right EMG | Test | P value |
| Delta pre-kainate (A) | Kruskal-Wallis test | P = 0.0017 |
| Baseline vs. day 1 | +Dunn's multiple comparison test | ns |
| Baseline vs. day 2 | +Dunn's multiple comparison test | ns |
| Baseline vs. day 3 | +Dunn's multiple comparison test | ** |
| Baseline vs. day 4 | +Dunn's multiple comparison test | * |
| Baseline vs. day 5 | +Dunn's multiple comparison test | ns |
| Delta post-kainate | Kruskal-Wallis test | P < 0.0001 |
| Baseline vs. day 1 | +Dunn's multiple comparison test | ns |
| Baseline vs. day 2 | +Dunn's multiple comparison test | ns |
| Baseline vs. day 3 | +Dunn's multiple comparison test | **** |
| Baseline vs. day 4 | +Dunn's multiple comparison test | ** |
| Baseline vs. day 5 | +Dunn's multiple comparison test | ** |
| Theta pre-kainate (B) | Kruskal-Wallis test | P = 0.0601 |
| Baseline vs. day 1 | +Dunn's multiple comparison test | ns |
| Baseline vs. day 2 | +Dunn's multiple comparison test | ns |
| Baseline vs. day 3 | +Dunn's multiple comparison test | * |
| Baseline vs. day 4 | +Dunn's multiple comparison test | ns |
| Baseline vs. day 5 | +Dunn's multiple comparison test | ns |
| Theta post-kainate | Kruskal-Wallis test | P = 0.0020 |
| Baseline vs. day 1 | +Dunn's multiple comparison test | ns |
| Baseline vs. day 2 | +Dunn's multiple comparison test | ns |
| Baseline vs. day 3 | +Dunn's multiple comparison test | * |
| Baseline vs. day 4 | +Dunn's multiple comparison test | ns |
| Baseline vs. day 5 | +Dunn's multiple comparison test | ns |
| Beta pre-kainate (C) | Kruskal-Wallis test | P = 0.0729 |
| Baseline vs. day 1 | +Dunn's multiple comparison test | ns |
| Baseline vs. day 2 | +Dunn's multiple comparison test | ns |
| Baseline vs. day 3 | +Dunn's multiple comparison test | ns |
| Baseline vs. day 4 | +Dunn's multiple comparison test | ns |
| Baseline vs. day 5 | +Dunn's multiple comparison test | ns |
| Beta post-kainate | Kruskal-Wallis test | P = 0.0019 |
| Baseline vs. day 1 | +Dunn's multiple comparison test | ns |
| Baseline vs. day 2 | +Dunn's multiple comparison test | ns |
| Baseline vs. day 3 | +Dunn's multiple comparison test | ns |
| Baseline vs. day 4 | +Dunn's multiple comparison test | ns |
| Baseline vs. day 5 | +Dunn's multiple comparison test | ns |
| Low gamma pre-kainate (D) | Kruskal-Wallis test | P = 0.7468 |
| Baseline vs. day 1 | +Dunn's multiple comparison test | ns |
| Baseline vs. day 2 | +Dunn's multiple comparison test | ns |
| Baseline vs. day 3 | +Dunn's multiple comparison test | ns |
| Baseline vs. day 4 | +Dunn's multiple comparison test | ns |
| Baseline vs. day 5 | +Dunn's multiple comparison test | ns |
| Low gamma post-kainate | Kruskal-Wallis test | P = 0.1812 |
| Baseline vs. day 1 | +Dunn's multiple comparison test | ns |
| Baseline vs. day 2 | +Dunn's multiple comparison test | ns |
| Baseline vs. day 3 | +Dunn's multiple comparison test | ns |
| Baseline vs. day 4 | +Dunn's multiple comparison test | ns |
| Baseline vs. day 5 | +Dunn's multiple comparison test | ns |
| High gamma pre-kainate (E) | Kruskal-Wallis test | P = 0.9133 |
| Baseline vs. day 1 | +Dunn's multiple comparison test | ns |
| Baseline vs. day 2 | +Dunn's multiple comparison test | ns |
| Baseline vs. day 3 | +Dunn's multiple comparison test | ns |
| Baseline vs. day 4 | +Dunn's multiple comparison test | ns |
| Baseline vs. day 5 | +Dunn's multiple comparison test | ns |
| High gamma post-kainate | Kruskal-Wallis test | P = 0.9786 |
| Baseline vs. day 1 | +Dunn's multiple comparison test | ns |
| Baseline vs. day 2 | +Dunn's multiple comparison test | ns |
| Baseline vs. day 3 | +Dunn's multiple comparison test | ns |
| Baseline vs. day 4 | +Dunn's multiple comparison test | ns |
| Baseline vs. day 5 | +Dunn's multiple comparison test | ns |
| Delta pre-kainate (A) |  |  |
| day 3 vs. day 4 | Mann Whitney test | P = 0.6523 |
| day 4 vs. day 5 | Mann Whitney test | P = 0.3008 |
| Delta post-kainate |  |  |
| day 3 vs. day 4 | Mann Whitney test | P = 0.1009 |
| day 4 vs. day 5 | Mann Whitney test | P = 0.6277 |
| Theta pre-kainate (B) |  |  |
| day 3 vs. day 4 | Mann Whitney test | P = 0.0742 |
| day 4 vs. day 5 | Mann Whitney test | P = 0.3008 |
| Theta post-kainate |  |  |
| day 3 vs. day 4 | Mann Whitney test | P = 0.325 |
| day 4 vs. day 5 | Mann Whitney test | P = 0.1167 |
| Beta pre-kainate (C) |  |  |
| day 3 vs. day 4 | Mann Whitney test | P = 0.3008 |
| day 4 vs. day 5 | Mann Whitney test | P > 0.9999 |
| Beta post-kainate |  |  |
| day 3 vs. day 4 | Mann Whitney test | P = 0.6621 |
| day 4 vs. day 5 | Mann Whitney test | P = 0.3765 |
| Low gamma pre-kainate |  |  |
| day 3 vs. day 4 | Mann Whitney test | P = 0.8203 |
| day 4 vs. day 5 | Mann Whitney test | P = 0.4961 |
| Low gamma post-kainate |  |  |
| day 3 vs. day 4 | Mann Whitney test | P = 0.9321 |
| day 4 vs. day 5 | Mann Whitney test | P = 0.5303 |
| High gamma pre-kainate |  |  |
| day 3 vs. day 4 | Mann Whitney test | P = 0.4961 |
| day 4 vs. day 5 | Mann Whitney test | P = 0.2500 |
| High gamma post-kainate |  |  |
| day 3 vs. day 4 | Mann Whitney test | P = 0.5940 |
| day 4 vs. day 5 | Mann Whitney test | P = 0.8223 |

Table 9. Left-right primary motor cortices coherence. Statistical data.

| TABLE 9 \| Interhemispheric coherence between primary motor cortices (Figure 6) | | | |
| --- | --- | --- | --- |
| Left – right motor cortices EcoG coherence | Test | | P value |
| Delta pre-kainate (A) | | Kruskal-Wallis test | P < 0.0001 |
| Baseline vs. day 1 | | +Dunn's multiple comparison test | ns |
| Baseline vs. day 2 | | +Dunn's multiple comparison test | *** |
| Baseline vs. day 3 | | +Dunn's multiple comparison test | ** |
| Baseline vs. day 4 | | +Dunn's multiple comparison test | **** |
| Baseline vs. day 5 | | +Dunn's multiple comparison test | ** |
| Delta post-kainate | | Kruskal-Wallis test | P = 0.0003 |
| Baseline vs. day 1 | | +Dunn's multiple comparison test | ns |
| Baseline vs. day 2 | | +Dunn's multiple comparison test | * |
| Baseline vs. day 3 | | +Dunn's multiple comparison test | **** |
| Baseline vs. day 4 | | +Dunn's multiple comparison test | ** |
| Baseline vs. day 5 | | +Dunn's multiple comparison test | * |
| Theta pre-kainate (B) | | Kruskal-Wallis test | P < 0.0001 |
| Baseline vs. day 1 | | +Dunn's multiple comparison test | ns |
| Baseline vs. day 2 | | +Dunn's multiple comparison test | ** |
| Baseline vs. day 3 | | +Dunn's multiple comparison test | ** |
| Baseline vs. day 4 | | +Dunn's multiple comparison test | *** |
| Baseline vs. day 5 | | +Dunn's multiple comparison test | *** |
| Theta post-kainate | | Kruskal-Wallis test | P = 0.0027 |
| Baseline vs. day 1 | | +Dunn's multiple comparison test | ns |
| Baseline vs. day 2 | | +Dunn's multiple comparison test | ns |
| Baseline vs. day 3 | | +Dunn's multiple comparison test | *** |
| Baseline vs. day 4 | | +Dunn's multiple comparison test | * |
| Baseline vs. day 5 | | +Dunn's multiple comparison test | ** |
| Beta pre-kainate (C) | | Kruskal-Wallis test | P < 0.0001 |
| Baseline vs. day 1 | | +Dunn's multiple comparison test | ns |
| Baseline vs. day 2 | | +Dunn's multiple comparison test | * |
| Baseline vs. day 3 | | +Dunn's multiple comparison test | * |
| Baseline vs. day 4 | | +Dunn's multiple comparison test | ** |
| Baseline vs. day 5 | | +Dunn's multiple comparison test | *** |
| Beta post-kainate | | Kruskal-Wallis test | P = 0.0295 |
| Baseline vs. day 1 | | +Dunn's multiple comparison test | ns |
| Baseline vs. day 2 | | +Dunn's multiple comparison test | ns |
| Baseline vs. day 3 | | +Dunn's multiple comparison test | * |
| Baseline vs. day 4 | | +Dunn's multiple comparison test | ns |
| Baseline vs. day 5 | | +Dunn's multiple comparison test | * |
| Low gamma pre-kainate (D) | | Kruskal-Wallis test | P = 0.1347 |
| Baseline vs. day 1 | | +Dunn's multiple comparison test | ns |
| Baseline vs. day 2 | | +Dunn's multiple comparison test | ns |
| Baseline vs. day 3 | | +Dunn's multiple comparison test | ns |
| Baseline vs. day 4 | | +Dunn's multiple comparison test | ns |
| Baseline vs. day 5 | | +Dunn's multiple comparison test | ns |
| Low gamma post-kainate | | Kruskal-Wallis test | P = 0.0891 |
| Baseline vs. day 1 | | +Dunn's multiple comparison test | ns |
| Baseline vs. day 2 | | +Dunn's multiple comparison test | ns |
| Baseline vs. day 3 | | +Dunn's multiple comparison test | ns |
| Baseline vs. day 4 | | +Dunn's multiple comparison test | ns |
| Baseline vs. day 5 | | +Dunn's multiple comparison test | ns |
| High gamma pre-kainate (E) | | Kruskal-Wallis test | P = 0.7715 |
| Baseline vs. day 1 | | +Dunn's multiple comparison test | ns |
| Baseline vs. day 2 | | +Dunn's multiple comparison test | ns |
| Baseline vs. day 3 | | +Dunn's multiple comparison test | ns |
| Baseline vs. day 4 | | +Dunn's multiple comparison test | ns |
| Baseline vs. day 5 | | +Dunn's multiple comparison test | ns |
| High gamma post-kainate | | Kruskal-Wallis test | P = 0.0014 |
| Baseline vs. day 1 | | +Dunn's multiple comparison test | ns |
| Baseline vs. day 2 | | +Dunn's multiple comparison test | ns |
| Baseline vs. day 3 | | +Dunn's multiple comparison test | ns |
| Baseline vs. day 4 | | +Dunn's multiple comparison test | ns |
| Baseline vs. day 5 | | +Dunn's multiple comparison test | ns |
| Delta pre-kainate (A) | |  |  |
| day 3 vs. day 4 | | Mann Whitney test | P = 0.5566 |
| day 4 vs. day 5 | | Mann Whitney test | P = 0.8203 |
| Delta post-kainate | |  |  |
| day 3 vs. day 4 | | Mann Whitney test | P = 0.1976 |
| day 4 vs. day 5 | | Mann Whitney test | P = 0.4812 |
| Theta pre-kainate (B) | |  |  |
| day 3 vs. day 4 | | Mann Whitney test | P = 0.3750 |
| day 4 vs. day 5 | | Mann Whitney test | P = 0.4961 |
| Theta post-kainate | |  |  |
| day 3 vs. day 4 | | Mann Whitney test | P = 0.2132 |
| day 4 vs. day 5 | | Mann Whitney test | P = 0.5793 |
| Beta pre-kainate (C) | |  |  |
| day 3 vs. day 4 | | Mann Whitney test | P = 0.7695 |
| day 4 vs. day 5 | | Mann Whitney test | P = 0.5703 |
| Beta post-kainate | |  |  |
| day 3 vs. day 4 | | Mann Whitney test | P = 0.7765 |
| day 4 vs. day 5 | | Mann Whitney test | P = 0.4652 |
| Low gamma pre-kainate (D) | |  |  |
| day 3 vs. day 4 | | Mann Whitney test | P = 0.7695 |
| day 4 vs. day 5 | | Mann Whitney test | P = 0.4258 |
| Low gamma post-kainate | |  |  |
| day 3 vs. day 4 | | Mann Whitney test | P = 0.4294 |
| day 4 vs. day 5 | | Mann Whitney test | P = 0.5793 |
| High gamma pre-kainate (E) | |  |  |
| day 3 vs. day 4 | | Mann Whitney test | P = 0.2754 |
| day 4 vs. day 5 | | Mann Whitney test | P = 0.9102 |
| High gamma post-kainate | |  |  |
| day 3 vs. day 4 | | Mann Whitney test | P = 0.0837 |
| day 4 vs. day 5 | | Mann Whitney test | P = 0.6239 |
